# The isolation and characterization of *Stenotrophomonas maltophilia* T4-like bacteriophage DLP6

**DOI:** 10.1371/journal.pone.0173341

**Published:** 2017-03-14

**Authors:** Danielle L. Peters, Paul Stothard, Jonathan J. Dennis

**Affiliations:** 1 6-065 Centennial Centre for Interdisciplinary Science, Department of Biological Sciences, University of Alberta, Edmonton, Alberta, Canada; 2 Department of Agricultural, Food and Nutritional Science, University of Alberta, Edmonton, Alberta, Canada; National Cancer Center, JAPAN

## Abstract

Increasing isolation of the extremely antibiotic resistant bacterium *Stenotrophomonas maltophilia* has caused alarm worldwide due to the limited treatment options available. A potential treatment option for fighting this bacterium is ‘phage therapy’, the clinical application of bacteriophages to selectively kill bacteria. Bacteriophage DLP6 (vB_SmoM-DLP6) was isolated from a soil sample using clinical isolate *S*. *maltophilia* strain D1571 as host. Host range analysis of phage DLP6 against 27 clinical *S*. *maltophilia* isolates shows successful infection and lysis in 13 of the 27 isolates tested. Transmission electron microscopy of DLP6 indicates that it is a member of the *Myoviridae* family. Complete genome sequencing and analysis of DLP6 reveals its richly recombined evolutionary history, featuring a core of both T4-like and cyanophage genes, which suggests that it is a member of the T4-superfamily. Unlike other T4-superfamily phages however, DLP6 features a transposase and ends with 229 bp direct terminal repeats. The isolation of this bacteriophage is an exciting discovery due to the divergent nature of DLP6 in relation to the T4-superfamily of phages.

## Introduction

The spread and increasing incidence of antibiotic resistance is imminent, with experts suggesting we will face a “post-antibiotic era” in the 21^st^ century [1]. The situation has recently become dire, as a new mechanism of antibiotic resistance towards one of the globally-recognized ‘last-resort’ antibiotics colistin has evolved [2]. The extremely antibiotic resistant bacterium *Stenotrophomonas maltophilia* has been increasingly identified as a causative agent in both nosocomial and community-acquired infections [3]. Infections associated with *S*. *maltophilia* include, but are not limited to pneumonia, bacteremia, meningitis, endocarditis, catheter-related bacteremia/septicemia and acute exacerbations in patients with cystic fibrosis and chronic obstructive pulmonary disease [3, 4]. Due to the ubiquitous nature of *S*. *maltophilia* in the environment and the possibility of spreading this bacterium through cough-generated aerosols, infection prevention has proven to be difficult [3, 5]. Once infected with *S*. *maltophilia*, treatment options are limited due to its innate resistance to a broad array of antibiotics including trimethoprim / sulfamethoxazole, β-lactams, macrolides, cephalosporins, fluoroquinolones, aminoglycosides, carbapenems, chloramphenicol, tetracyclines, and polymyxin. Due to the limited treatment options, alternative strategies are being investigated in order to combat this extremely drug resistant bacterium.

The clinical application of bacteriophages to selectively kill infecting bacteria, known as “phage therapy”, is a potential solution to extremely antibiotic resistant bacteria. During the Second World War, Soviet and German armies utilized phage therapy to treat dysentery of their soldiers, while the United States military conducted classified research on it [6]. Additionally, some clinicians worldwide continued to use phage therapy from the 1920s to the early 1950s [6]. Unfortunately, with the advent of broad-spectrum antibiotics and a limited understanding of phage biology, phage therapy was largely abandoned in the West. However, with the recent significant rise in antibiotic resistance in bacterial pathogens throughout the world, interest in the efficacious use of phage therapy has been renewed. Recent studies utilizing phage therapy to treat multi-drug resistant infections in animal models [7–16] and human clinical trials [17–20] have shown that phages can be a successful treatment option. To use phages in the clinical treatment of infections, the U.S. Food and Drug Administration requires characterization of the phages to prove they do not include moronic genes encoding toxins or other undesirable proteins which could enhance bacterial virulence [21]. Therefore, all phages isolated for use in clinical therapy must be fully characterized through complete genome sequencing and functional analysis. Towards that goal, the isolation and characterization of the novel *S*. *maltophilia* phage vB_SmoM-DLP6 (DLP6) is described herein. This phage is related to T4-superfamily of phages and exhibits an interesting combination of T4 and cyanobacteriophage genes.

## Materials and methods

### Bacterial strains and growth conditions

Five *S*. *maltophilia* strains were acquired from the Canadian *Burkholderia cepacia* complex Research and Referral Repository (CBCCRRR; Vancouver, BC). The *S*. *maltophilia* strains used for isolation of phage from soil samples were D1585, D1571, D1614, D1576 and D1568. An additional 22 *S*. *maltophilia* strains were gifted from the Provincial Laboratory for Public Health—North (Microbiology), Alberta Health Services, for host range analysis. All strains were grown aerobically overnight at 30°C on half-strength Luria-Bertani (½ LB) solid medium or in ½ LB broth with shaking at 225 RPM.

### Phage isolation, propagation, host range analysis and electron microscopy

DLP6 was isolated from planter soil located at the Kinsman Sports Center in Edmonton, Alberta, Canada using strain D1571 and a previously described extraction protocol [22]. Propagation of DLP6 was performed using soft agar overlays: 100 μl liquid culture and 100 μl phage stock were incubated 20 min at room temperature, mixed with 3 ml 0.7% ½ LB top agar, overlaid on a plate of ½ LB solid medium, and incubated at 30°C until plaque formation was complete. High titre stocks were made by overlaying plates with confluent lysis with 3 ml modified SM and the top agar was scraped into a sterile falcon tube. The top agar was pelleted by centrifugation for 5 min at 10,000 × g and the supernatant was removed and filter-sterilized using a Millex-HA 0.45 μm syringe-driven filter unit (Millipore, Billerica, MA), followed by storage at 4°C. Titres were obtained using serial dilutions of phage stock into SM, followed by the soft agar overlay technique described above and incubation at 30°C until plaque formation was complete.

Host range analysis was performed using a panel of 27 clinical *S*. *maltophilia* and 19 *P*. *aeruginosa* strains. Soft-agar overlays containing 100 μl liquid culture were allowed to solidify for 10 minutes at room temperature. Plates were spotted with 10 μl drops of DLP6 at multiple dilutions and assayed for clearing and/or plaque formation after incubation at 30°C for 36 h.

For electron microscopy, phage stocks were prepared as described above with the following modifications: ½ LB agarose plates and ½ LB soft agarose were used for overlays, MilliQ-filtered water for phage recovery and a 0.22 μm filter was used for syringe-driven filtration. A carbon-coated copper grid was incubated with lysate for 2 min and stained with 4% uranyl acetate for 30 s. Transmission electron micrographs were captured using a Philips/FEI (Morgagni) transmission electron microscope with charge-coupled device camera at 80 kV (University of Alberta Department of Biological Sciences Advanced Microscopy Facility). The average capsid diameter, tail length and tail width were calculated using Microsoft Excel based on measurements from 10 individual virions.

### Phage DNA isolation, RFLP analysis and sequencing

DLP6 genomic DNA was isolated from bacteriophage lysate using the Wizard Lambda DNA purification system (Promega Corp., Madison, WI) with a modified protocol [23, 24]. A 10 ml aliquot of high-titre filter-sterilized phage lysate was treated with 10 μl DNase I (Thermo Scientific, Waltham, MA), 100 μl 100x DNase I buffer (1 M Tris–HCl, 0.25 M MgCl2, 10 mM CaCl2), and 6 μl RNase (Thermo Scientific) and incubated 1 h at 37°C to degrade the bacterial nucleic acids. Following incubation, 400 μl of 0.5 M EDTA and 25 μl of 20 mg/ml proteinase K (Applied Biosystems, Carlsbad, CA) was added and incubated 1 h at 55°C to inactivate DNase I. The lysate was cooled to room temperature and added to 8.4 g of guanidine thiocyanate, along with 1 ml of 37°C resuspended Wizard DNA Clean-Up Resin (Promega Corporation, Madison, WI). This mixture was rocked for 10 min then pelleted by centrifugation for 10 min at 5,000 x g. The supernatant was drawn off until ~5 ml remained. Remaining mixture was resuspended by swirling, transferred to a syringe attached to a Wizard Minicolumn (Promega Corporation). The Wizard Minicolumn was attached to The Vac-Man^®^ Jr. Laboratory Vacuum Manifold (Promega Corporation) and placed under vacuum to remove the supernatant. The column was then washed with 2 ml 80% isopropanol and dried by centrifugation for 2 min at 10,000 x g. Phage DNA was eluted from the column following a 1 min incubation of 100 μl of 80°C nuclease-free water (Integrated DNA Technologies, Coralville, IA) followed by centrifugation for 1 min at 10,000 x g. A NanoDrop ND-1000 spectrophotometer (Thermo Scientific, Waltham, MA) was used to determine purity and concentration of eluted DNA.

Restriction fragment length polymorphism (RFLP) analysis was used with 18 FastDigest (Thermoscientific) restriction enzymes: BamHI, EcoRI, AciI, HpaII, XbaI, HindIII, KpnI, SmaI, ApaI, SaII, PstI, SpHI, SacI, ClaI, Ndel, SpeI, Xhol and HaeIII. Restriction reactions were set up using 1 μl of FastDigest enzyme, 2 μl of FastDigest restriction buffer, 1 μg of phage DNA and topped up to 20 μl with nuclease free water. Reactions were incubated at 37°C for 20 min and separated on a 1% (wt/vol) agarose gel in 1x TAE (pH 8.0). Sequencing of DLP6 was performed at The Applied Genomics Core at the University of Alberta. Purified DLP6 DNA was prepared for sequencing using a Nextera XT library prep kit, creating a library size of 223 bp. The library was used for paired-end sequencing on a MiSeq (Illumina, San Diego, CA) platform using a MiSeq v2 reagent kit. The Q30 for all reads was 92.5%.

### Lifecycle of DLP6

DLP6 resistant colonies of *S*. *maltophilia* D1571 were isolated by using the top agar overlay method using a phage stock at a titer of 1x10^5^ PFU/mL. Following overnight incubation at 30°C, a 3 mL aliquot of SM was transferred to each plate, supernatant was collected and used for serial dilutions to obtain superinfection resistant single colonies on ½ LB plates. Individual colonies were picked, washed with SM and used to produce freezer stocks. Superinfection experiments were performed using overnight cultures of the potential lysogens and DLP6 at a 1x10^5^ PFU/mL titer. Single colonies for each potential lysogen or pseudolysogen were used for colony PCR to detect the presence of DLP6. Identifying the temporary presence of DLP6 in the cell during pseudolysogeny was determined using specific sets of internal primers for DLP6.

### Pulse Field Gel Electrophoresis (PFGE)

Overnight cultures of wild type D1571 and five DLP6 PCR positive single colony isolates were used to generate plugs following the protocol outline by Sueh *et al*. 2013[25]. A SpeI (New England Biolabs) or XbaI (Thermo Fisher Scientific) restriction digest was set up for all six samples and incubated for 45 min at 37°C. A 1% agarose gel made with TAE was used for the PFGE. Switch time was 50–90 for 12 hrs at 6 V/cm. Ladders used were *Saccharomyces cerevisiae* and Lambda DNA/HindIII markers (Promega) for comparison of high and low molecular weight DNA. Gels were stained using SYBR Safe DNA Gel Stain (Thermo Fisher Scientific) and visualized with a ChemiDoc MP imaging system and the Image Lab software (Bio-Rad).

### Bioinformatics analysis

A single contig was assembled using the CLC Genomics Workbench (Qiagen, Toronto, ON). Although no ambiguous regions in the contig were observed, PCR amplification and sequencing was used to confirm the assembly. All attempts by PCR amplification to identify a DNA segment between the direct repeats or to show genome circularization via direct repeat annealing were negative. Open reading frames (ORFs) were identified with the GLIMMER plugin [26] for Geneious [27] using the Bacteria and Archaea setting, as well as GeneMarkS (http://exon.gatech.edu/GeneMark/genemarks.cgi) for phage [28] and Prodigal [29]. Conserved domain searches were performed using CD-Search [30]. Pfam was used to identify functions for hypothetical protein hits from BLASTP [31]. The contig was annotated and confirmed with an Interactive Remote Invocation Service utilizing the RAST pipeline [32–34]. BLASTN and BLASTP (for full genomes and individual proteins, respectively) was used to gain more information for each RAST annotation, and to identify any potential related phages [35]. BLASTP hits above 1.00E^-3^ were not recorded and the coding sequence (CDS) was annotated as hypothetical. Rho independent terminators were predicted using ARNold [36–38] searching both strands. Promoters were predicted using PHIRE [39] and plotted using WebLogo 3 [40]. tRNAs were identified using tRNAscan-SE using the general tRNA model [41]. Multiple sequence alignments were performed with the top 250 BLASTP hits for gp20 and from core T4 and cyanophage proteins [42] using the MUSCLE [43] plugin for Geneious [44]. The maximum number of iterations selected was 8, with the anchor optimization option selected. The trees from iterations 1 and 2 were not retained. The distance measure for iteration 1 was kmer6_6, and was pctid_kimura for subsequent iterations. The clustering method was UPGMB for all iterations. An unrooted tree was constructed from MUSCLE alignments with the FastTree 2.1.5 [45] plugin for Geneious [44]. The Jones-Taylor-Thornton model was used with rate category of sites set to 20. A PROmer comparison was conducted with DLP6 and ΦM12 with the following parameters: breaklen = 60, maxgap = 30, mincluster = 10, minmatch = 3 [46].

## Results and discussion

### Isolation, host range and morphology

**Fig 1:**
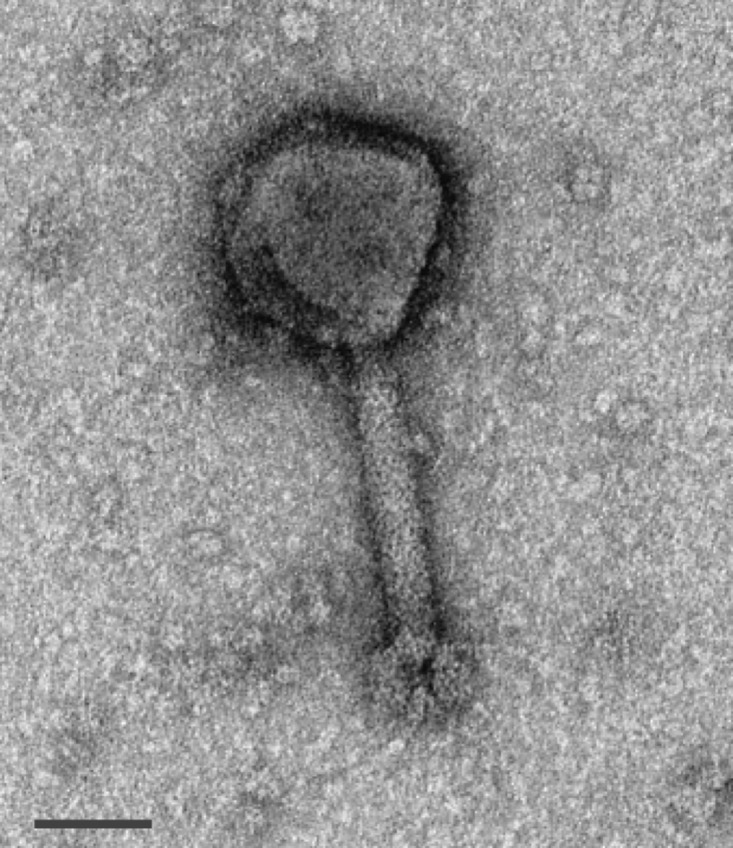
DLP6 phage morphology. Liquid phage lysate was incubated on a carbon coated copper grid, stained with 4% uranyl acetate and visualized at 180,000-fold magnification by a transmission electron microscope. Scale bar represent 50 nm. The average capsid height measurement for DLP6 was 99 nm, average tail length of 144 nm and average tail width of 23 nm.

Phage DLP6 was isolated from a soil sample using clinical isolate *S*. *maltophilia* strain D1571 as the host. Propagation of DLP6 to high titre has proven difficult in liquid cultures, with liquid grown lysate concentrations remaining constant at 10^6^ PFU/ml despite attempts to increase progeny numbers. DLP6 exhibits a unique plaquing inhibition that was previously observed in *Burkholderia cepacia* complex phages KL1 and AH2 [47], as well as *S*. *maltophlila* phages DLP1 and DLP2 [22]. Although high titer stocks (10^10^ plaque forming units [PFU]/ml) can easily be obtained using the top agar plating method, use of such high titre stock inhibits the plaque formation on a bacterial lawn. Plaquing of DLP6 is inhibited at titers above 10^6^ plaque forming units (PFU/ml). Plaque development occurs readily at 30°C within 24 hr, forming diffuse plaques with irregular boarders and a mean size of 0.8 ± 0.3 mm. Host range analysis of DLP6 revealed a moderate host range within *S*. *maltophilia* clinical isolates, infecting 13 out of 27 clinical isolates (S1 Table). Whereas *S*. *maltophilia* phages DLP1 and DLP2 exhibited some cross-species infectivity ^22^, extended host range analysis of DLP6 using *P*. *aeruginosa* isolates did not yield successful infections. Initially, we produced evidence to suggest that DLP6 existed in *S*. *maltophilia* D1571 as a prophage. However, after extensive experimentation it was determined that DLP6 undergoes only pseudolysogeny. PFGE analysis using SpeI or XbaI separately showed no integration of the DLP6 genome into the *S*. *maltophilia* D1571 chromosome, and DLP6-specific PCR indicated the genome’s presence after 2–3 passages, but not after >5 passages. DLP6 is classified in the *Myoviridae* family of the *Caudovirales* order due to its icosahedral head and contractile tail (Fig 1). The average capsid height, tail length and width measurements for DLP6 are 99, 144, and 23 nm respectively.

### Genome characterization

**Fig 2:**
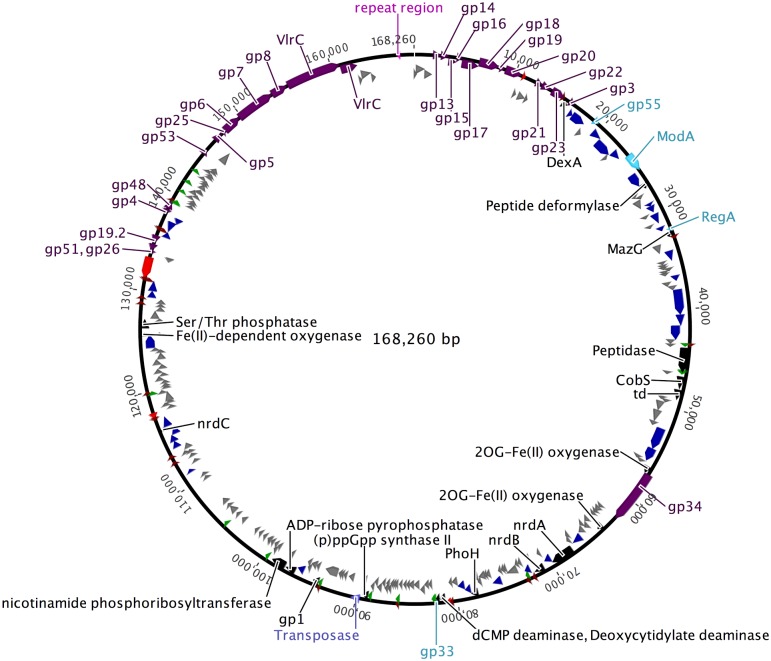
Genomic map of DLP6. The scale (in bp) is shown in the outermost periphery of the genome along with late viral promoters (dark green) and terminators (dark red), as predicted by the algorithims in the software programs PHIRE and ARNold, respectively. Assigned functions for each predicted open reading frame are as follows: auxiliary metabolism (black), lysis (red), gene expression (light blue), phage morphogenesis (dark purple), DNA replication/repair (lilac), tRNA (bright green), repeat region (pink) and hypothetical (grey). Due to space constraints, genes are located either inside of or outside the circle thereby reducing overlap. The bp numbering of the circular map takes into account annealing of the direct repeats, reducing the genome length from 168,489 bp (- 229 bp) to 168,260 bp.

Purified DLP6 gDNA was isolated and exposed to a panel of 18 restriction enzymes for RFLP analysis. Even though controls were performed to detect the presence of restriction inhibitors, the DLP6 genome was assembled into a linear scaffold of 168,489 bp with a GC content of 55.8% using 43,112 reads for a mean coverage of 57 reads and an overall Q30 score of 93.1%. The direct terminal repeats are covered by up to 45 reads (S1 Fig). The contig can be found in GenBank with the accession number KU682439.2. The genome is predicted to encode 241 coding DNA sequences and 30 tRNAs of 14 different specificities (Fig 2, S2 and S3 Tables). The genome is arranged in a semi-modular format, with DNA replication/repair genes (dark blue) primarily grouped together, whereas the regulatory genes (light blue) are mainly grouped within the same region as the DNA replication/repair genes. Auxiliary metabolism (black) genes are dispersed throughout the genome, occurring in small pairs rather than a large set. The phage morphogenesis (dark purple) genes are grouped in a cluster, with the exception of gp34 (AAY80_073; long tail fiber). The 30 tRNA genes are grouped together spanning the genome from 92,962–113,834 bp. There is no lysis (red) module in DLP6, rather four genes encoding typical lysis proteins are randomly located throughout the genome.

Three interesting phage-encoded proteins are ADP-ribosyltransferases (Alt: AAY80_209, ModA: AAY80_029, ADP-ribosyltransferase: AAY80_145) that were identified using the Pfam database. Two of these proteins are orthologs to T4 proteins Alt and ModA. It is known that phage T4 encodes three ADP-ribosyltransferases (Alt, ModA and ModB), each modifying specific groups of host proteins. The Alt protein is a component of the phage head and enters the host cell during the infection process with the phage DNA, ModA and ModB [48]. Following entry into the cell, Alt immediately ADP-ribosylates the host RNA polymerase (RNAP), causing transcription of host genes to stop and transcription from the T4 “early” promoters to be carried out instead [49]. The ModA modification of the host RNAP prevents transcription from T4 early promoters and possibly primes the RNAP for T4-encoded auxiliary factors (gp55, gp33, gp45 and gp44/62) to transcribe middle and late genes [50]. Identification of the previously hypothetical proteins into the Alt and ModA families helps to provide insight into the possible role these proteins play in DLP6 phage infection and transcription initiation and regulation.

**Fig 3:**
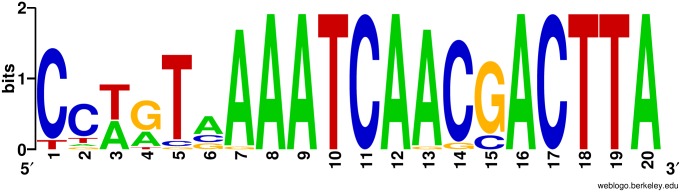
Predicted promoter sequence in DLP6. Putative phage promoter consensus sequence identified using PHIRE [39] and plotted using WebLogo 3 [40].

Phage promoter sequences lack the conserved structure observed in bacterial promoters with -10 and -35 regions; instead, they feature short consensus sequences that are specific to different phages [51]. These short consensus sequences were identified using the phage-specific program PHIRE, and visualized with WebLogo 3 (Fig 3). There are 25 phage promoters identified, with 22 of the phage promoters found repeating in groups of two in front of a gene cluster. A single phage promoter is located upstream of genes AAY80_058 (hypothetical protein) and AAY80_059 (peptidase protein). The next single phage promoter is located upstream of the gene cluster beginning with AAY80_139 (kinase protein) through to AAY80_150 (hypothetical protein). The final single phage promoter is located upstream of a small cluster of hypothetical proteins beginning at AAY80_217 through to AAY80_220. Two phage promoters are found upstream of AAY80_060 (hypothetical protein) in a gene cluster coding for 32 proteins, including DNA primase (AAY80_083) phage tail fiber (AAY80_073), ribonucleotide diphosphate reductase subunit alpha (AAY80_086) and beta (AAY80_089), and many hypothetical proteins (locus tags ending in 060, 063–067, 070–071, 074–077, 079–082, 084–085, 088 and 090). The next set of phage promoters is located upstream of AAY80_092 (hypothetical protein) to AAY80_112 (hypothetical protein). Annotated genes included in this gene cluster are AAY80_098 (RNase H), AAY80_104 (PhoH), AAY80_106 (exonuclease), AAY80_109 (SleB), AAY80_110 (dCMP deaminase) and AAY80_111 (deoxycytidylate deaminase). The next two sets of gene clusters, AAY80_113–120 and AAY80_121–130, encode hypothetical proteins only and both clusters are under control of two promoters each. Gene cluster AAY80_131–138 utilizes two promoter sequences and encodes mainly hypothetical proteins, but also a guanosine 3',5'-bis(diphosphate) 3'-pyrophosphohydrolase (AAY80_131) and a transposase (AAY80_133). The adjacent gene cluster under control of two promoters is upstream of AAY80_151 (hypothetical protein) through to AAY80_158 (hypothetical protein) and contains nine tRNAs. The remaining 21 tRNAs are under control of two promoters upstream of AAY80_159 (hypothetical protein) through to AAY80_180 (hypothetical protein). Two promoters control the next gene cluster spanning from AAY80_181–202 that contains genes encoding many hypothetical proteins and proteins such as DNA ligase (AAY80_192), DNA helicase loader (AAY80_201) and ssDNA binding protein (AAY80_202). Two small gene clusters encoding a total of eight hypothetical proteins (AAY80_213–216 and AAY80_221–224) are both under control of two phage promoters. The last set of double phage promoters controls the gene cluster from AAY80_225 to AAY80_057 where the phage genome is circularized. This final gene cluster contains many proteins involved in DNA replication and homologous recombination.

Rho-independent transcription termination sites were discovered using ARNold, which utilizes two complementary programs, Erpin [36] and RNAmotif [37]. Of the 54 potential termination sites identified, only 19 were retained as authentic because they had a ΔG of -10 kcal/mol or less (Table 1). The 19 terminators were found downstream of AAY80_018 (precursor of major head subunit), AAY80_042 (triphosphate pyrophosphohydrolase), AAY80_057 (hypothetical protein), AAY80_090 (hypothetical protein), AAY80_091 (RuvC), AAY80_108 (hypothetical protein), AAY80_120 (hypothetical protein), AAY80_138 (hypothetical protein), AAY80_166 (hypothetical protein), AAY80_168 (hypothetical protein), AAY80_175 (endolysin), AAY80_180 (hypothetical protein), AAY80_199 and AAY80_200 (hypothetical proteins), AAY80_202 (ssDNA binding protein), AAY80_203 (lysozyme), AAY80_208 (endonuclease protein), AAY80_209 (hypothetical protein) and AAY80_212 (baseplate tail tube cap protein).

**Table 1:** Predicted Rho-independent terminators in DLP6. Rho-independent terminators were identified using the ARNold [36, 37] program and putative terminators with a ΔG value of -10 kcal/mol or less were retained. DNA that is predicted to form the loop in the RNA is in emboldened, whereas DNA that is predicted to encode an RNA stem is underlined.

Start	Program	Strand	Sequence	-ΔG
15290	both	+	CAATAAGAGAAGCCGCC**GCAA**GGCGGCTTTT	16.80
32949	both	+	AAGCCAGACAAGCCCCAGG**CTCCGC**CCTGGGGCTTTT	17.50
43682	both	+	TCAACGACTTAGCCCCAGA**CCCCGT**TCTGGGGCTTTT	16.10
72171	both	+	CTAATTGGAAAGCCGCCTCCGGGCGGCTTT	13.40
72816	Rnamotif	+	GACTTAGCGAAGCCCCC**GCCT**GGGGGCTTT	13.50
80627	both	+	TTGATGGAAAAGGCTCTCT**GCGGCTAACCGGCTC**AGAGAGCCTTT	13.46
85964	both	+	GACTTAGCGAAGCCCCGC**CCT**GCGGGGCTTT	14.80
93801	both	+	CGCCCCTGGAAGCCCGCTT**GGTCCGAGTGACT**AGGCGGGCTTTT	13.32
112696	both	+	GTAATCCAAAAGGGCTGGT**GTCCAAGAT**GCCAGCCCTTTT	15.70
113321	Rnamotif	+	CTATCTCGAAAGCCGCC**GCAA**GGCGGCTTT	16.80
117336	Rnamotif	-	ATCCATCTCGTCGAGAAGC**AGGTCTTCCT**GCTTCTCGTTTT	11.70
119975	both	+	TCAACGACTTAGCCCCTGA**GCCACC**TCAGGGGCTTTTCATTCCTG	17.10
128844	both	+	CTGTGAGAAAAGCCCTGCC**TTGATC**GGCGGGGCTTTTCCTTTGAT	17.60
129411	Erpin	+	CTTGACCGAAAAGCCCCGAAAGGGGCTTTTCTTTTGCCCA	14.90
131176	both	-	GCAAAGAAAAAGCCCAGGC**ATTGC**GCCTGGGCTTTTCAATTACAT	18.20
131178	both	+	GTAATTGAAAAGCCCAGGC**GCAAT**GCCTGGGCTTTTTCTTTGCG	17.80
136291	both	-	GATACAAGAAAGGCTCCCT**CT**CGGGAGCCTTTTGCTTTCACT	15.00
136293	both	+	TGAAAGCAAAAGGCTCCC**GAGA**GGGAGCCTTTCTTGTATCA	17.80
139414	Rnamotif	+	GACTTAGCGAAGCCCCC**GAAA**GGGGGCTTTACTTTTGGG	17.60

### Phylogeny using DLP6 gp20 protein

**Fig 4:**
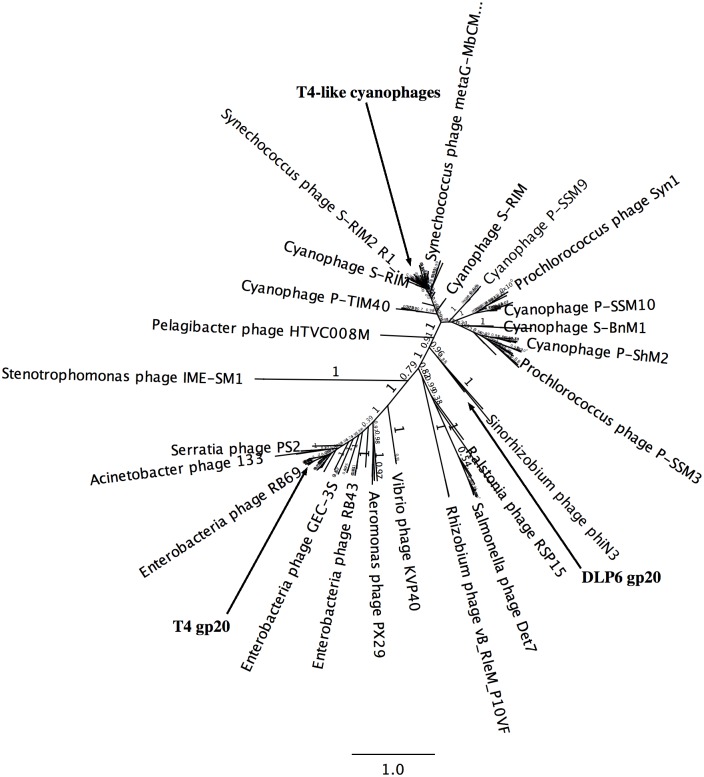
Unrooted gp20 (portal vertex protein: AAY80_011) tree. FastTree was used to generate the tree from a MUSCLE alignment between DLP6 gp20 and the top 250 BLASTP sequences. The local support value for each branch is shown on the tree and the bar is a marker of branch distance length. The clades featuring gp20 of T4 and gp20 of T4-superfamily cyanophages are indicated.

A BLASTN search using DLP6 genome as a query revealed the closest hits are *Sinorhizobium* phages ΦN3, ΦM19, ΦM7 and ΦM12. All four phages have coverage of only 4% with a 72% identity. Although initial BLASTP and BLASTN searches indicated DLP6 was more closely related to members of the T4-superfamily of phages, more comparisons were required to classify DLP6 as a T4-superfamily phage. For a commonly used phylogenetic comparison, the protein sequence of portal protein gp20 was used in a BLASTP search to identify 250 of the most similar sequences [52–54]. The most significant hits came from cyanophages grouped into the T4-superfamily. A MUSCLE alignment was completed using the 250 BLASTP results compared to the DLP6 gp20 protein (AAY80_011). This alignment was then used to generate an unrooted tree with the FastTree plugin for Geneious (Fig 4). The generated tree positions DLP6 in a clade with *Sinorhizobium* phages ΦM12, ΦN3 and *Caulobacter* phage Cr30 (Fig 4).

### DLP6 contains features from T4-superfamily enteric bacteriophages and T4-superfamily cyanophages

**Fig 5:**
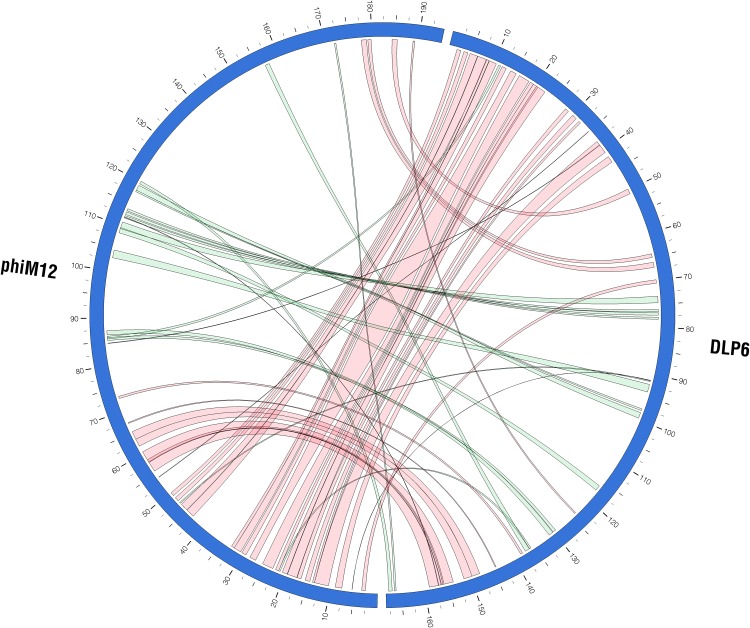
Circos plot of DLP6 and ΦM12 PROmer comparisons. Green ribbons indicate regions of similarity between the two genomes at the protein level encoded on the same strand, and representing a similarity of greater than 32%, with an average similarity of 56%. Red ribbons indicate regions of similarity at the protein level encoded on opposite strands, representing a similarity of greater than 32%, with an average similarity of 56%. The scale (in kbp) is shown on the periphery of the plots. PROmer parameters: breaklen = 60, maxgap = 30, mincluster = 10, minmatch = 3.

Genomic organization of DLP6 is similar to the *Sinorhizobium* transducing phage ΦM12, which has been classified into a new T4-superfamily fusing features of cyanophages and phages of enteric bacteria [42] (Fig 5). A set of “core universal” and “nearly universal” proteins has been determined for T4-superfamily phages [55, 56]. DLP6 contains all of the core and nearly universal proteins common to all T4-superfamily phages (Table 2). A MUSCLE comparison was used to align the T4-superfamily phage core proteins against their respective orthologs from 18 T4-superfamily phages, and their percent identity to each ortholog was determined (Table 2). Similar to ΦM12, DLP6 contains a second copy of gp19 tail tube monomer that was not found in the 17 other phages studied (21% similarity). The order of the gene products presented in Table 2 corresponds to the order they are found within the DLP6 genome. This order differs from the T4-superfamily phages, which start with gp41 (DNA primase-helicase). Results from the MUSCLE alignment reveal that DLP6 core proteins are most similar to their orthologs from cyanophages, with the exception of the UvsX protein, which is most similar to T4-superfamily phages of enteric bacteria. The alignment also shows DLP6 has the highest percent identity to ΦM12 core proteins. The ortholog with the highest similarity to a DLP6 protein was ΦM12 gp23, with 65% similarity. Overall, the most highly conserved proteins between DLP6 and the T4-superfamily cyanophages are RegA (early transcriptional regulator) and gp23 (major capsid protein), with similarity rates averaging 59 and 57% respectively. This suggests that DLP6 is divergent from the T4-superfamily phages. DLP6 does share additional proteins that are found within the T4-superfamily cyanophages or the T4-superfamily enteric phages.

**Table 2:** MUSCLE alignment percent identity score of full-length protein sequences of DLP6 against universal core and nearly universal core proteins of 18 T4-superfamily phages. Numbers indicate percent similarity to the related DLP6 protein.

**Gene product: function**	**Cyanophages**												**Enteric phages**					
	**ΦM12**	**S-SM2**	**P-SSM4**	**P-Syn1**	**HTVC008M**	**S-Syn9**	**S-Syn19**	**S-ShM2**	**P-Syn33**	**S-SM1**	**P-HM1**	**S-CRM01**	**T4**	**Aeh**	**44 RR**	**KVP 40**	**RB43**	**ΦW-14**
**gp13:** neck protein AAY80_004	53.2	35.5	34.2	34.3	39.1	36.1	35.7	33.5	35.7	37.2	36.1	22.9	21.7	19.4	20.2	23.1	25.2	22.6
**gp14:** neck protein AAY80_005	43.9	17	28	28.8	26.3	24.4	27.2	23.2	22.8	28.6	18.3	20.4	24.7	26.7	24.8	27.7	24.5	24.8
**gp15:** proximal tail sheath stabilization AAY80_006	40	31.9	27.9	32	31.5	33.2	27.3	34.8	34.1	33.3	27	30.9	24.3	26.8	23.5	16.6	23.6	20.8
**gp16:** small terminase AAY80_007	30.7	27.6	25.9	25.5	21.4	29.2	27.5	31.1	26.7	30.4	30.4	30.1	21.7	18.6	16.7	17.6	23.2	15.2
**gp17:** large terminase AAY80_008	42.5	47.2	48.1	47.2	46.9	48.5	48.7	48.9	49	48.8	46.1	42.8	27.8	28.5	27.7	29.8	29.8	32
**gp18:** tail sheath monomer AAY80_009	48.4	33.3	37.3	35	43.7	35.7	38.1	13.5	37.6	38.8	34.4	32.8	31.7	31.8	30.9	31.5	31.8	21.9
**gp19:** tail tube monomer AAY80_010	46.8	40.1	33.5	34.2	32.6	33.5	35.3	28.2	33.7	34	30.1	40.7	33.3	30.6	31.8	36.8	34.9	13.8
**gp20:** portal vertex protein AAY80_011	46.1	46.7	47.7	46.6	43.2	49.1	49.1	46.7	46.7	49.6	46.2	40.7	33.4	32	33.7	36	32.7	31.2
**gp21:** prohead core scaffold/protease AAY80_016	41.3	49.3	47.7	54.1	51.6	49.1	50	49.1	47.7	49.1	49.5	51.1	32.1	32.5	32.8	34.6	32.4	30
**gp22:** scaffold prohead core protein AAY80_017	22.3	29.1	26.5	29.9	30.1	26.5	26.5	26.6	27	25.9	27.8	25.9	19.3	20.2	22.1	17.8	20.6	11.6
**gp23:** precursor of major head subunit AAY80_018	65.4	59.6	55.7	52.5	60.5	57.1	58	56.3	58	55.8	55.9	53.8	34.4	33.3	34.8	35	34.3	44.8
**DexA:** exonuclease AAY80_019		32.5	32.1	35.7	30.5		33.3	35	34.2	32.9	29.3	22.2	12.2	13.7	11.2	8.2	14.6	11.4
**gp3:** head proximal tip of tail tube AAY80_020	27.3	31.5	29.1	31.4	18.5	29.3	27.6	28.3	29.2	30.9	29.4	27.2	20	18.5	17.8	17.5	20.3	14.8
**UsvY:** recombination, repair ssDNA binding AAY80_021	39.6	27.1	30	22.4	29.2	27.9	27.2	31.9	26.6	27.7	28.5	27.8	20.8	18.1		20.8	16.7	19.7
**UsvW:** helicase AAY80_022	40	42.9	44.8	41.1	47.9	47.1	44.8	47.2	45.8	45.5	36.8	39.5	32.3	33.9	32.1	36	31.4	30.7
**gp55:** sigma factor, late transcription AAY80_024	36.6	37.3	43.5	42.5	41.6	40.7	40.4	44.1	38.4	42.3	42.5	37.8	23.9	24.8	22	25.4	22.8	24.7
**gp47:** recombination endonuclease subunit AAY80_025	42.7	33.9	36.7	34.3	39.4	39.2	40.3	36.2	35.5	39.8	37.7	38.5	27.5	23.1	24.9	27.1	28.2	22.4
**Gene product: function**	**Cyanophages**												**Enteric phages**					
	**ΦM12**	**S-SM2**	**P-SSM4**	**P-Syn1**	**HTVC008M**	**S-Syn9**	**S-Syn19**	**S-ShM2**	**P-Syn33**	**S-SM1**	**P-HM1**	**S-CRM01**	**T4**	**Aeh**	**44 RR**	**KVP 40**	**RB43**	**ΦW-14**
**gp46:** recombination endonuclease subunit AAY80_026	45.7	42.9	43.4	43.2	44.3	44.3	45.9	45	44.9	45.2	44.2	37.8	31.2	25.5	29.2	28	31.3	20.5
**gp45**: sliding clamp accessory protein AAY80_036	34.2	43.8	40.1	38.6	42.2	33.5	40.8	35.9	38.8	39.6	40.6	39	28	27	26.9	36.9	26.1	25.8
**gp44:** clamp loader subunit AAY80_038	46.8	55.7	54.1	48.9	52.9	56.2	54.3	55.3	54.6	55.3	52.4	48.1	34.4	36.6	35.3	35.7	32.5	32.8
**gp62:** clamp loader subunit AAY80_040	37.1	31.8	29.6	32.1	35.6	33.1	31.6	42.5	33.8	31.6	37.2	31.8	21.1	18.1	19.5	27.3	21	25.5
**RegA:** early gene translational repressor AAY80_041	52.2	60	60	59.2	59.2	60	62.3	60.8	60	60.8	60	56.2	49.6	48.4	48.4	48.4	41.6	33.1
**gp43:** DNA polymerase AAY80_054	39.7	42.2	41.1	40	38	42.6	41.1	40.9	41	42.6	41.9	39.6	28.7	31.3	18.8	31.3	29.8	26.4
**UsvX:** recombination protein AAY80_055	47.9	23.9	25.3	26.4	25.1	25.3	25.3	24.5	24.7	26.1	25.8	26.2	49.9	52.7		47.8	46.1	35.1
**gp41:** DNA primase-helicase AAY80_056	50.5	57	58.8	53.7	54.6	53.5	53.7	56.2	53.5	54.3	53.8	51.7	37.4	40.9	39.7	38.6	38.7	31
**Td:** thymidylate synthase AAY80_062	11.4	12.5	11.3	12.5		10.9	14.4	12.3	11.6	11.9	11.4	10.6	11.4	9.3	10.8	7.4	12.1	8.8
**gp61:** DNA primase AAY80_083	44.6	34	35.8	37.8	37	39.8	36.2	37.5	39.2	40.8	39	37.5	31.1	32.4	33.4	31.6	30.9	30.6
**NrdA:** ribonucleotide reductase subunit A AAY80_086		46.8	46.3	47.4	45.5	47.7	47.3	46.5	48.1	47.9	47.9	48.1	43.6	29.9	17	44.3	43.8	13.1
**NrdB:** ribonucleotide reductase subunit B AAY80_089		44.2	45.9	42.6	44.6	45.1	44	44.5	41.2	44.5	44	41.2	37.5	39.3	18.3	41.7	37.7	19.4
**gp33:** late promoter transcription factor AAY80_112	18.7	23.3	22.7	27.4	21.3	23.8	24.4	22.6	22.4	26.2	29.9	26.7	22.7	19	24.1	20.7	14.8	30.2
**NrdC:** glutaredoxin AAY80_173	39	29.5	29.5	30.9	32.2	25.4	30.8	29.5	28.2	28.2	25.6	29.1	36.8	27.3		25.3	34.4	
**gp59:** DNA helicase loader AAY80_201	34.6	32.5	35.5		29.1	34.1	35.2	28.4	35.5	35	31.2	30.1	23.4	24.8	19.3	21.7	20.5	
**gp32:** ssDNA binding protein AAY80_202	48.8	48.3	46.7	44.5	44.7	46.5	47.1	48	49.5	48.5	47.3	46.7	34.2	30.2	30.6	33.1	25.3	
**gp51:** baseplate hub assembly catalyst AAY80_205	39.6	49.1	37.3	34.9	28.9	33.3	38.6	48.3	31.8	35.6	39.6	31	7.1		8.1	22.7	5.5	9.7
**gp26:** baseplate hub AAY80_206	36.3	31.1	34.3	32.1	36.1	35.4	36.3	34.4	35.6	38.9	31	27.2	19.1	23	15.8	24.6	14.8	24.3
**Gene product: function**	**Cyanophages**												**Enteric phages**					
	**ΦM12**	**S-SM2**	**P-SSM4**	**P-Syn1**	**HTVC008M**	**S-Syn9**	**S-Syn19**	**S-ShM2**	**P-Syn33**	**S-SM1**	**P-HM1**	**S-CRM01**	**T4**	**Aeh**	**44 RR**	**KVP 40**	**RB43**	**ΦW-14**
**gp4:** head completion protein AAY80_211	50.3	46.3	45.3	35.1	38.3	47.3	46.6	44.6	45.9	45.3	46.6	30	38.2	38.2	47.4	39.2	37.9	36.9
**gp48:** baseplate tail tube cap AAY80_212	19	14.6	17	15.6	15.9	17.4	12.9	19.8	19.8	15.5	12.8	15.8	15.5	14.7	14.2	13.1	11.1	22.8
**gp53:** baseplate wedge AAY80_230	29.8	21	20.9	17	23.6	23.5	20.1	23.8	24.4	22.4	15.5	21.8	16.1	19.7	20.8	20.2	18	20.9
**gp5:** baseplate hub and tail lysozyme AAY80_232	11.2	9.4	9.6	5.8	22.2	8.7	8.7	9.2	9	8.6	7.2	8.7	16.7	15.9	15.7	22.3	13.8	10.3
**gp25:** baseplate wedge AAY80_233	44	27.8	35.1	33.8	36.6	30.8	29.1	28.4	29.9	26.1	27.1	30.1	25.8	27.9	25.6	27.3	28.8	25.2
**gp6:** baseplate wedge AAY80_234	33.1	26.7	24.3	30.5	31.6	24.7	24.7	23.1	25.1	23.5	29.6	27.3	21.5	21.6	23.6	21.8	22.7	22.6
**gp7:** baseplate wedge initiator AAY80_235	11.3	5.3	4	6.3	7.4	4.7	4.5	4.4	4.3	4.7	4.8	4.4	11.1	10.8	10.5	10.1	11.1	
**gp8:** baseplate wedge AAY80_236	20.1	16.4	19.6	14.4	18.3	19.3	19.7	19.7	19.1	20.3	17.4	18.4	10.5	12.1	11	10.9	11.5	

All sequenced T4-superfamily cyanophages feature an accessory core set of 25 gene clusters (T4-GCs) that are not found within enteric bacteria T4-superfamily phages [55]. Of this accessory core, DLP6 encodes six of the core proteins: CobS (porphyrin biosynthetic protein), PhoH (P-starvation inducible protein), T4-CG 313 (hypothetical protein), T4-GC 321 (hypothetical protein), VlrC (predicted structural protein) and MazG (pyrophosphatase) (Table 3). Although DLP6 does contain these T4-superfamily cyanophage accessory core proteins, the DLP6 proteins are again divergent, with the maximum similarity found with the PhoH (P-starvation inducible protein) at 45% similar to P-HM1 cyanophage.

**Table 3:** MUSCLE alignment percent identity score of DLP6 amino acid sequences against T4-superfamily cyanophage accessory core proteins. DLP6 contains six of the 25 T4-superfamily cyanophage core proteins. Numbers indicate percent similarity to the related DLP6 protein.

Gene product: function	Cyanophages											
	ΦM12	S-SM2	P-SSM4	Syn1	HTVC008M	Syn9	Syn19	S-ShM2	Syn33	S-SM1	P-HM1	S-CRM01
**CobS:** porphyrin biosyntheic protein AAY80_061		41.6	41.9	43.6	40.3	42.2	41.2	41.9	41.5	41.1	42.7	40.7
**PhoH:** P-starvation inducible protein AAY80_104	33.0	42.5	44.4	44.7	42.8	44.0	43.8	42.2	43.0	42.6	44.8	39.2
**T4 Gc 313:** hypothetical protein AAY80_099	33.5	30.6	31.2	36.0	33.1	30.5	31.9	31.4	30.5	31.9	26.4	28.1
**T4 Gc 321:** hypothetical protein AAY80_103	39.2	25.3	17.7	20.7	30.4	24.1	25.3	25.3	17.7	24.1	21.0	14.3
**VlrC:** predicted structural protein AAY80_237	19.3	14.7	16.3	13.0	17.5	16.2	16.4	15.5	16.3	15.8	14.9	13.7
**MazG:** pyrophosphatase AAY80_042		16.9	16.4	20.6		16.3	19.4	16.9	16.3	18.1	16.9	16.3

DLP6 was found to contain nine out of the designated 30 non-cyanophage core T4-superfamily proteins. A MUSCLE alignment of these nine proteins indicates that although this phage does contain the proteins, they do not share high amino acid similarities (Table 4). The protein sharing the highest similarity was the RnaseH protein, which had a maximum similarity of 32.6% with the RB43 phage protein. Again, these results demonstrate that DLP6, although classified within the T4-superfamily, it is more divergent than the other accepted members.

**Table 4:** MUSCLE alignment percent identity score of DLP6 amino acid sequences against T4-superfamily non-cyanophage core proteins. This table includes 10 of the 32 non-cyanophage core proteins. Numbers indicate percent similarity to the related DLP6 protein.

Gene product: function	Enteric phages					
	T4	Aeh	44 RR	KVP 40	RB43	ΦW-14
**RnaseH** AAY80_098	28.9	32.5	28.9	32.2	32.6	8.8
**gp1:** dNMP kinase AAY80_139	12.9	10.8	10	12.3	8.9	9.2
**dCMP deaminase** AAY80_110	24.5	24.6	22.8	26.6	18	16
**DsbA:** ssDNA binding protein AAY80_035	28.9	20.5	20.5	22.9	25.3	
**gp34:** long tail fiber AAY80_073	11.9	11.6	12.1	11.6	11.9	
**gp30:** dna ligase AAY80_192	24.7	23.5	21.9	29.7	24.5	32.3
**Tk:** thymidine kinase AAY80_139	13.5	13.5	12	11.4	13.5	8.4
**GroES:** head assembly chaperone with GroEL AAY80_164	16.7	12.8	14	20	7.6	
**gp59:** loader of gp41 DNA helicase AAY80_201	23.4	24.8	19.3	21.7	20.5	

Differences between the T4-superfamily superfamily members discussed in this paper as compared to DLP6 are interesting, given that DLP6 contains the complete set of T4-superfamily core group of proteins, six accessory core cyanophage proteins, and nine non-cyanophage accessory core proteins. Moreover, the ends of DLP6 genome feature 229 bp direct terminal repeats, unlike the genomes of other members of the T4-superfamily of phages which are cyclically permuted. This finding is unusual, and suggests that DNA circularization in the host cell occurs at cohesive sites. The direct terminal repeats are located in a region of DNA devoid of ORFs, or homology to known DNA sequences. The average number of tRNAs encoded by the T4-superfamily phages is ten, with the exception of S-CRM01, a freshwater cyanophage that encodes 33 tRNAs [57]. The DLP6 genome contains 30 tRNAs, which is in the high range for the T4-superfamily. DLP6 also encodes a transposase (AAY80_133) (Fig 2, S2 Table).

## Conclusions

*S*. *maltophilia* bacteriophage DLP6 was isolated from a soil sample using *S*. *maltophilia* strain D1571 as the host bacterium. Phage DLP6 exhibits a moderate host range, infecting 13 out of 27 clinical *S*. *maltophilia* strains. A phylogenetic comparison of gp20 portal protein against the top 250 BLASTP results places DLP6 in a clade with *Sinorhizobium* phages ΦM12, ΦN3 and *Caulobacter* phage Cr30. Although DLP6 does encode all of the T4-superfamily core and nearly universal core orthologs, the similarity between these proteins and their nearest neighbors is typically less than 60%. The DLP6 genome also encodes six T4-superfamily cyanophage core proteins, but again, the nearest neighbor similarity is below 40%. There are nine T4-superfamily non-cyanophage core proteins found within the DLP6 genome, though the similarity between the DLP6 proteins and the T4-superfamily enteric phage orthologs averaged less than 30% similarity. Unlike other T4-superfamily phages, DLP6 possesses 229 bp direct terminal repeats at the ends of its genome instead of circular permutation. Although DLP6 also encodes a transposon, experimental investigation has shown it does not form a stable prophage in *S*. *maltophilia* strain D1571. The results presented in this paper suggest DLP6 is a divergent T4-superfamily phage, exhibiting characteristics not yet identified in other T4-superfamily phages.

## Supporting information

S1 Fig Illumina pair-end sequencing reads aligning to start (1A) and end (1B) of DLP6 contig. Geneious was used to map R1 and R2 to DLP6 reference contig. Sensitivity was set to medium sensitivity/fast. Fine-tuning was iterated up to five times. Reads were not trimmed. Coverage at ends was between one and 45.
(PDF)

S1 Table Host range determination of DLP6. (DOCX)

S2 Table Bacteriophage DLP6 coding sequences. (DOCX)

S3 Table DLP6 tRNA annotations. (DOCX)
