# Supplementary figures and images for: The isolation and characterization of Stenotrophomonas maltophilia T4-like bacteriophage DLP6
Source: PLoS One. 2017 Mar 14;12(3):e0173341. doi: 10.1371/journal.pone.0173341 (PMC5349666; doi:10.1371/journal.pone.0173341)

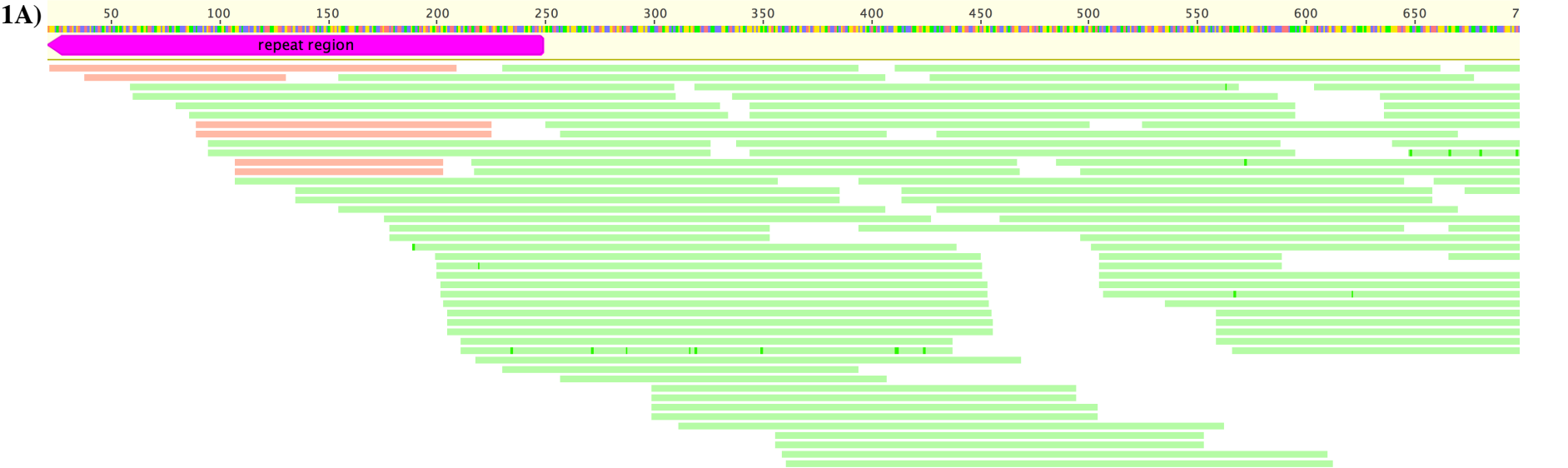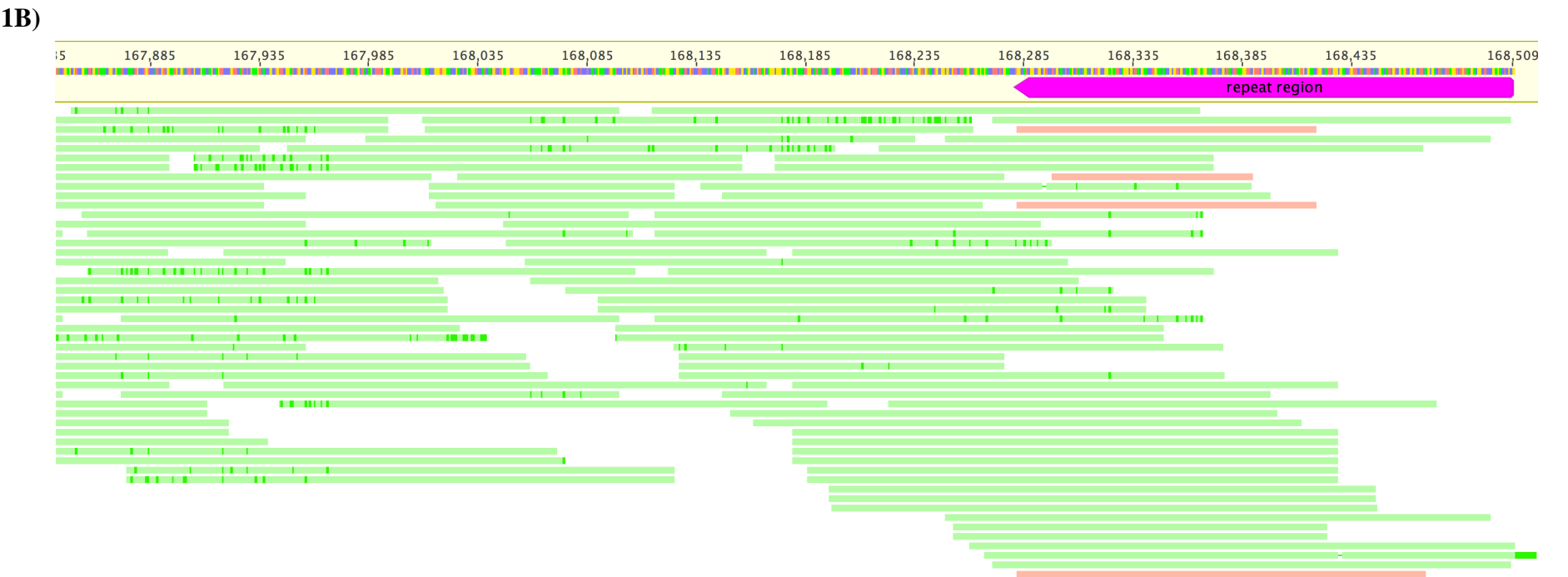

Supplement: S1 Fig — Geneious was used to map R1 and R2 to DLP6 reference contig. Sensitivity was set to medium sensitivity/fast. Fine-tuning was iterated up to five times. Reads were not trimmed. Coverage at ends was between one and 45. (PDF) [file pone.0173341.s001.pdf]
