# Supplementary material for: The isolation and characterization of Stenotrophomonas maltophilia T4-like bacteriophage DLP6
Source: PLoS One. 2017 Mar 14;12(3):e0173341. doi: 10.1371/journal.pone.0173341 (PMC5349666; doi:10.1371/journal.pone.0173341)
Supplement: S1 Table — (DOCX) [file pone.0173341.s002.docx]

| ***S. maltophilia* strain** | **DLP6** | ***P. aeruginosa* strain** | **DLP6** |
| --- | --- | --- | --- |
| 101^c^ | - | PA01 | – |
| 102^c^ | + | HER1004 | – |
| 103^c^ | + | HER1012 | – |
| 152^c^ | - | 14715 | – |
| 155^c^ | +++ | Utah3 | – |
| 174^c^ | - | Utah4 | – |
| 176^c^ | + | 14655 | – |
| 213^c^ | +++ | 6106 | – |
| 214^c^ | - | pSHU-OTE | – |
| 217^c^ | ++ | D1606D^a,b^ | – |
| 218^c^ | - | D1615C^a,b^ | – |
| 219^c^ | + | D1619M^a,b^ | – |
| 230^c^ | + | D1620E^a,b^ | – |
| 236^c^ | - | D1623C^a,b^ | – |
| 242^c^ | - | ENV003^a^ | – |
| 249^c^ | - | ENV009^a^ | – |
| 278^c^ | - | FC0507^a^ | – |
| 280^c^ | - | R285 | – |
| 282^c^ | - | 14672 | – |
| 287^c^ | ++ |  |  |
| 446^c^ | + |  |  |
| 667^c^ | ++ |  |  |
| D1585^a,b^ | - |  |  |
| D1571^a,b^ | +++ |  |  |
| D1614^a,b^ | - |  |  |
| D1576^a,b^ | ++ |  |  |
| D1568^a,b^ | - |  |  |
| ^a^ Obtained from the Canadian *Burkholderia cepacia* complex Research Referral Repository. | | | |
| ^b^ Cystic fibrosis patient isolate.  ^c^ Isolates from the Provincial Laboratory for Public Health - North (Microbiology), Alberta Health Services. | | | |
| –, No sensitivity to phage; +, plaques at 10^-2^; ++, clearing at 10^-2^; +++, plaques at 10^-4^; ++++, plaques at 10^-6^. | | | |
