# Supplementary material for: The isolation and characterization of Stenotrophomonas maltophilia T4-like bacteriophage DLP6
Source: PLoS One. 2017 Mar 14;12(3):e0173341. doi: 10.1371/journal.pone.0173341 (PMC5349666; doi:10.1371/journal.pone.0173341)
Supplement: S2 Table — (DOCX) [file pone.0173341.s003.docx]

**Supplementary Table 2. Bacteriophage DLP6 coding sequences.**

| **CDS** | **Start** | **End** | **Length (AA)** | **Strand** | **Start Codon** | **Putative Function** | **Closest relative** | **E-Value** | **Source** | **Version** |
| --- | --- | --- | --- | --- | --- | --- | --- | --- | --- | --- |
|  |  |  |  |  |  |  |  |  |  |  |
| 1 | 1,664 | 1,837 | 57 | + | ATG | hypothetical protein |  |  |  |  |
| 2 | 1,821 | 2,699 | 292 | + | ATG | hypothetical protein | hypothetical protein | 2.0E-05 | Φpp2 | [WP_018251053.1](http://www.ncbi.nlm.nih.gov/protein/517062235?report=genbank&log$=prottop&blast_rank=1&RID=AD4UTJAW015) |
| 3 | 2,696 | 3,448 | 250 | + | ATG | hypothetical protein |  |  |  |  |
| 4 | 3,483 | 4,223 | 246 | + | ATG | gp13: neck protein | neck protein | 3.0E-101 | Cr30 | [YP_009098960.1](http://www.ncbi.nlm.nih.gov/protein/725949367?report=genbank&log$=prottop&blast_rank=1&RID=ACZR631S01R) |
| 5 | 4,227 | 4,982 | 251 | + | ATG | gp14: neck protein | neck protein | 3.0E-62 | ΦN3 | [YP_009212340.1](http://www.ncbi.nlm.nih.gov/protein/971758990?report=genbank&log$=prottop&blast_rank=1&RID=ACZR631S01R) |
| 6 | 4,982 | 5,785 | 267 | + | ATG | gp15: proximal tail sheath stabilization | proximal tail sheath stabilization | 2.0E-68 | ΦM12 | [YP_009143012.1](http://www.ncbi.nlm.nih.gov/protein/828805651?report=genbank&log$=prottop&blast_rank=1&RID=ACZR631S01R) |
| 7 | 5,782 | 6,300 | 172 | + | ATG | g16: small terminase subunit | small terminase subunit | 4.0E-22 | S-SM1 | [YP_004323004.1](http://www.ncbi.nlm.nih.gov/protein/326782624?report=genbank&log$=prottop&blast_rank=1&RID=ACZR631S01R) |
| 8 | 6,272 | 7,936 | 554 | + | ATG | gp17: large terminase subunit | large terminase subunit | 0.0E+00 | ACG-2014c | [YP_007001827.1](http://www.ncbi.nlm.nih.gov/protein/418487485?report=genbank&log$=prottop&blast_rank=1&RID=ACZR631S01R) |
| 9 | 7,999 | 10,014 | 671 | + | ATG | gp18: tail sheath protein | tail sheath protein | 0.0E+00 | Cr30 | [YP_009098945.1](http://www.ncbi.nlm.nih.gov/protein/725949352?report=genbank&log$=prottop&blast_rank=1&RID=ACZR631S01R) |
| 10 | 10,035 | 10,568 | 177 | + | ATG | gp19: tail tube protein | tail tube protein | 1.0E-51 | ΦM12 | [YP_009142981.1](http://www.ncbi.nlm.nih.gov/protein/828805620?report=genbank&log$=prottop&blast_rank=1&RID=ACZR631S01R) |
| 11 | 10,636 | 12,303 | 555 | + | ATG | gp20: portal vertex of head | protal vertex of head protein | 0.0E+00 | S-SM1 | [YP_004323020.1](http://www.ncbi.nlm.nih.gov/protein/326782608?report=genbank&log$=prottop&blast_rank=1&RID=ACZR631S01R) |
| 12 | 12,329 | 12,490 | 53 | + | ATG | hypothetical protein |  |  |  |  |
| 13 | 12,490 | 12,747 | 85 | + | ATG | holin protein | holin | 6.0E-03 | JCL1032 | [YP_007002991.1](http://www.ncbi.nlm.nih.gov/protein/418489136?report=genbank&log$=prottop&blast_rank=1&RID=ACZR631S01R) |
| 14 | 12,750 | 13,535 | 261 | + | ATG | hypothetical protein |  |  |  |  |
| 15 | 13,538 | 13,855 | 105 | + | ATG | hypothetical protein |  |  |  |  |
| 16 | 13,855 | 14,517 | 220 | + | ATG | gp21: prohead core scaffold and protease | prohead core scaffold and protease | 3.0E-75 | S-PM2 | [YP_195140.1](http://www.ncbi.nlm.nih.gov/protein/58532917?report=genbank&log$=prottop&blast_rank=1&RID=ACZR631S01R) |
| 17 | 14,565 | 15,578 | 337 | + | ATG | gp22: scaffold prohead core | scaffold prohead core | 9.0E-55 | uvMED | [BAR30798.1](http://www.ncbi.nlm.nih.gov/protein/787059083?report=genbank&log$=prottop&blast_rank=1&RID=ACZR631S01R) |
| 18 | 15,618 | 16,919 | 433 | + | ATG | gp23: precursor of major head subunit | precursor of major head subunit | 0.0E+00 | ΦN3 | [YP_009212304.1](http://www.ncbi.nlm.nih.gov/protein/971758954?report=genbank&log$=prottop&blast_rank=1&RID=ACZR631S01R) |
| 19 | 17,030 | 17,623 | 197 | + | GTG | DexA | exonuclease | 3.0E-13 | 44RR2.8t | [NP_932376.1](http://www.ncbi.nlm.nih.gov/protein/37651502?report=genbank&log$=prottop&blast_rank=1&RID=ACZR631S01R) |
| 20 | 17,660 | 18,169 | 169 | + | ATG | gp3: tail tube protein | phage tail tube protein | 2.0E-32 | uvMED | [BAR32912.1](http://www.ncbi.nlm.nih.gov/protein/787061632?report=genbank&log$=prottop&blast_rank=1&RID=ACZR631S01R) |
| 21 | 18,185 | 18,619 | 144 | + | ATG | UvsY | putative repair and recombination protein | 4.0E-33 | ΦM12 | [YP_009142965.1](http://www.ncbi.nlm.nih.gov/protein/828805605?report=genbank&log$=prottop&blast_rank=1&RID=ACZR631S01R) |
| 22 | 18,623 | 20,086 | 487 | + | GTG | UvsW | UvsW | 2.0E-157 | uvMED | [BAR36206.1](http://www.ncbi.nlm.nih.gov/protein/787065492?report=genbank&log$=prottop&blast_rank=1&RID=ACZR631S01R) |
| 23 | 20,083 | 20,469 | 128 | + | GTG | hypothetical protein |  |  |  |  |
| 24 | 20,579 | 21,211 | 210 | + | ATG | gp55: sigma factor late transcription | hypothetical protein | 3.0E-48 | P-RSM6 | [YP_007675119.1](http://www.ncbi.nlm.nih.gov/protein/472341603?report=genbank&log$=prottop&blast_rank=1&RID=ACZR631S01R) |
| 25 | 21,247 | 22,335 | 362 | + | ATG | gp47: recombination endonuclease subunit | recombination endonuclease subunit | 3.0E-106 | ΦN3 | [YP_009212290.1](http://www.ncbi.nlm.nih.gov/protein/971758940?report=genbank&log$=prottop&blast_rank=1&RID=ACZR631S01R) |
| 26 | 22,332 | 24,047 | 571 | + | GTG | gp46: recombination endonuclease | recombination endonuclease subunit | 6.0E-176 | S-ShM2 | [YP_004322802.1](http://www.ncbi.nlm.nih.gov/protein/326782402?report=genbank&log$=prottop&blast_rank=1&RID=ACZR631S01R) |
| 27 | 24,044 | 24,238 | 64 | + | ATG | hypothetical protein | polyprotein | 8.0E-04 | ATROP06/BR/2012 | [AKI82132.1](http://www.ncbi.nlm.nih.gov/protein/823752063?report=genbank&log$=prottop&blast_rank=1&RID=ACZR631S01R) |
| 28 | 24,235 | 25,098 | 287 | + | ATG | hypothetical protein |  |  |  |  |
| 29 | 25,452 | 27,143 | 563 | + | ATG | ModA | hypothetical protein | 8.0E-19 | RSL1 | [YP_001950115.1](http://www.ncbi.nlm.nih.gov/protein/189427016?report=genbank&log$=prottop&blast_rank=1&RID=ACZR631S01R) |
| 30 | 27,179 | 28,525 | 448 | + | ATG | SNF2 DNA repair protein | SNF2 DNA repair protein | 4.0E-122 | Cr30 | [YP_009098887.1](http://www.ncbi.nlm.nih.gov/protein/725949294?report=genbank&log$=prottop&blast_rank=1&RID=ACZR631S01R) |
| 31 | 28,522 | 29,079 | 185 | + | ATG | peptide deformylase | peptide deformylase | 4.0E-43 | Cr30 | [YP_009098904.1](http://www.ncbi.nlm.nih.gov/protein/725949311?report=genbank&log$=prottop&blast_rank=1&RID=ACZR631S01R) |
| 32 | 29,079 | 29,429 | 116 | + | ATG | hypothetical protein | hypothetical protein | 1.0E-13 | *Gemmata sp.* IIL30 | [WP_052559698.1](http://www.ncbi.nlm.nih.gov/protein/918689602?report=genbank&log$=prottop&blast_rank=1&RID=AD4UTJAW015) |
| 33 | 29,429 | 29,731 | 100 | + | ATG | hypothetical protein |  |  |  |  |
| 34 | 29,740 | 30,597 | 285 | + | ATG | hypothetical protein | hypothetical protein | 1.0E-121 | Cr30 | [YP_009098901.1](http://www.ncbi.nlm.nih.gov/protein/725949308?report=genbank&log$=prottop&blast_rank=1&RID=ACZR631S01R) |
| 35 | 30,619 | 30,861 | 80 | + | ATG | DsbA dsDNA binding protein | DsbA dsDNA binding protein | 3.0E-05 | T4 | [NP_049858.1](http://www.ncbi.nlm.nih.gov/protein/9632736?report=genbank&log$=prottop&blast_rank=1&RID=ACZR631S01R) |
| 36 | 30,877 | 31,548 | 223 | + | ATG | gp45: sliding clamp | sliding clamp | 3.0E-67 | P-SSM2 | [YP_214389.1](http://www.ncbi.nlm.nih.gov/protein/61806029?report=genbank&log$=prottop&blast_rank=1&RID=ACZR631S01R) |
| 37 | 31,598 | 31,771 | 57 | + | ATG | hypothetical protein |  |  |  |  |
| 38 | 31,764 | 32,699 | 311 | + | ATG | gp44: sliding clamp loader subunit | clamp loader subunit | 7.0E-130 | S-MbCM25 | AHB80773.1 |
| 39 | 32,696 | 32,929 | 77 | + | ATG | hypothetical protein |  |  |  |  |
| 40 | 33,166 | 33,600 | 144 | + | GTG | gp62: clamp loader subunit | clamp loader subunit | 3.0E-33 | ΦM12 | [YP_009142948.1](http://www.ncbi.nlm.nih.gov/protein/828805588?report=genbank&log$=prottop&blast_rank=1&RID=ACZR631S01R) |
| 41 | 33,597 | 33,989 | 130 | + | ATG | regA | regA | 2.0E-54 | S-IOM18 | [YP_008126450.1](http://www.ncbi.nlm.nih.gov/protein/514051170?report=genbank&log$=prottop&blast_rank=1&RID=ACZR631S01R) |
| 42 | 33,989 | 34,405 | 138 | + | ATG | MazG | triphosphate pyrophosphohydrolase | 4.0E-23 | *Moumouvirus goulette* | [AGF85421.1](http://www.ncbi.nlm.nih.gov/protein/451927543?report=genbank&log$=prottop&blast_rank=1&RID=ACZR631S01R) |
| 43 | 34,789 | 35,745 | 318 | + | ATG | hypothetical protein | hypothetical protein | 2.0E-82 | S13 | [YP_009196385.1](http://www.ncbi.nlm.nih.gov/protein/971741889?report=genbank&log$=prottop&blast_rank=1&RID=ACZR631S01R) |
| 44 | 35,745 | 36,674 | 309 | + | ATG | N-acetyltransferase | N-acetyltransferase | 2.0E-08 | *Alistipes inops* | [WP_052131127.1](http://www.ncbi.nlm.nih.gov/protein/917524710?report=genbank&log$=prottop&blast_rank=1&RID=AD4UTJAW015) |
| 45 | 36,667 | 36,897 | 76 | + | ATG | hypothetical protein |  |  |  |  |
| 46 | 37,045 | 37,290 | 81 | + | ATG | hypothetical protein | hypothetical protein | 8.0E-07 | Lu11 | [YP_006382802.1](http://www.ncbi.nlm.nih.gov/protein/388684922?report=genbank&log$=prottop&blast_rank=1&RID=ACZR631S01R) |
| 47 | 37,301 | 37,525 | 74 | + | ATG | hypothetical protein |  |  |  |  |
| 48 | 37,522 | 37,737 | 71 | + | ATG | hypothetical protein | hypothetical protein | 1.0E-07 | uvMED | [BAR33778.1](http://www.ncbi.nlm.nih.gov/protein/787062653?report=genbank&log$=prottop&blast_rank=1&RID=ACZR631S01R) |
| 49 | 37,737 | 37,886 | 49 | + | ATG | hypothetical protein |  |  |  |  |
| 50 | 37,890 | 38,150 | 86 | + | ATG | hypothetical protein |  |  |  |  |
| 51 | 38,223 | 38,837 | 204 | + | ATG | sulfotransferase | sulfotransferase | 2.0E-28 | *Lysobacter* | [WP_055903507.1](http://www.ncbi.nlm.nih.gov/protein/947130344?report=genbank&log$=prottop&blast_rank=1&RID=AD4UTJAW015) |
| 52 | 38,845 | 39,264 | 139 | + | ATG | hypothetical protein |  |  |  |  |
| 53 | 39,261 | 39,680 | 139 | + | ATG | hypothetical protein | hypothetical protein | 4.0E-38 | *Janthinobacterium lividum* | [WP_010396923.1](http://www.ncbi.nlm.nih.gov/protein/498082767?report=genbank&log$=prottop&blast_rank=1&RID=AD4UTJAW015) |
| 54 | 39,682 | 42,228 | 848 | + | ATG | gp43: DNA polymerase | DNA polymerase | 0.0E+00 | syn9 | [YP_717843.1](http://www.ncbi.nlm.nih.gov/protein/113200680?report=genbank&log$=prottop&blast_rank=1&RID=ACZR631S01R) |
| 55 | 42,267 | 43,361 | 364 | + | ATG | UvsX | recombination protein | 3.0E-173 | IME-SM1 | [AKO61686.1](http://www.ncbi.nlm.nih.gov/protein/857290237?report=genbank&log$=prottop&blast_rank=1&RID=ACZR631S01R) |
| 56 | 43,361 | 44,788 | 475 | + | ATG | gp41: DNA primase/helicase | DNA primase/helicase | 0.0E+00 | S-IOM18 | [YP_008126466.1](http://www.ncbi.nlm.nih.gov/protein/514051186?report=genbank&log$=prottop&blast_rank=1&RID=ACZR631S01R) |
| 57 | 44,785 | 45,309 | 174 | + | ATG | hypothetical protein | hypothetical protein | 2.0E-06 | Cr30 | [YP_009098845.1](http://www.ncbi.nlm.nih.gov/protein/725949252?report=genbank&log$=prottop&blast_rank=1&RID=ACZR631S01R) |
| 58 | 45,427 | 45,597 | 56 | + | ATG | hypothetical protein |  |  |  |  |
| 59 | 45,607 | 48,036 | 809 | + | ATG | peptidase | peptidase | 2.0E-75 | P-RSM4 | [YP_004323283.1](http://www.ncbi.nlm.nih.gov/protein/326782885?report=genbank&log$=prottop&blast_rank=1&RID=ACZR631S01R) |
| 60 | 48,172 | 48,441 | 89 | + | ATG | hypothetical protein |  |  |  |  |
| 61 | 48,452 | 49,732 | 426 | + | ATG | CobS | porphyrin biosynthetic protein | 9.0E-119 | ACG-2014i | [YP_009140910.1](http://www.ncbi.nlm.nih.gov/protein/827047475?report=genbank&log$=prottop&blast_rank=1&RID=ACZR631S01R) |
| 62 | 49,818 | 50,792 | 324 | + | ATG | Td | FAD-dependent thymidylate synthase | 8.0E-120 | VCM | [CUR44270.1](http://www.ncbi.nlm.nih.gov/protein/952977557?report=genbank&log$=prottop&blast_rank=1&RID=ACZR631S01R) |
| 63 | 50,789 | 51,145 | 118 | + | GTG | hypothetical protein | hypothetical protein | 1.0E-18 | 7-7-1 | [YP_007006492.1](http://www.ncbi.nlm.nih.gov/protein/435844376?report=genbank&log$=prottop&blast_rank=1&RID=ACZR631S01R) |
| 64 | 51,138 | 51,320 | 60 | + | ATG | hypothetical protein |  |  |  |  |
| 65 | 51,394 | 52,311 | 305 | + | ATG | hypothetical protein | hypothetical protein | 3.0E-68 | ΦM12 | [YP_009143274.1](http://www.ncbi.nlm.nih.gov/protein/828805913?report=genbank&log$=prottop&blast_rank=1&RID=ACZR631S01R) |
| 66 | 52,316 | 53,551 | 411 | + | ATG | hypothetical protein | hypothetical protein | 2.0E-87 | HTVC008M | [YP_007517993.1](http://www.ncbi.nlm.nih.gov/protein/460042563?report=genbank&log$=prottop&blast_rank=1&RID=ACZR631S01R) |
| 67 | 53,580 | 53,993 | 137 | + | ATG | hypothetical protein |  |  |  |  |
| 68 | 54,039 | 56,207 | 722 | + | ATG | cytitidyltransferase | cytitidyltransferase | 9.0E-37 | ACG-2014a | [AIX45294.1](http://www.ncbi.nlm.nih.gov/protein/723041607?report=genbank&log$=prottop&blast_rank=1&RID=ACZR631S01R) |
| 69 | 56,234 | 57,535 | 433 | + | ATG | gp49: exodeoxyribonuclease VII large subunit | exodeoxyribonuclease VII large subunit | 2.0E-03 | *Thioalkalimicrobium aerophilum* | [WP_006460299.1](http://www.ncbi.nlm.nih.gov/protein/493505862?report=genbank&log$=prottop&blast_rank=1&RID=AD4UTJAW015) |
| 70 | 57,535 | 57,687 | 50 | + | ATG | hypothetical protein |  |  |  |  |
| 71 | 57,687 | 57,998 | 103 | + | ATG | hypothetical protein | hypothetical protein | 3.0E-05 | uvMED | [BAR32939.1](http://www.ncbi.nlm.nih.gov/protein/787061665?report=genbank&log$=prottop&blast_rank=1&RID=ACZR631S01R) |
| 72 | 58,009 | 58,653 | 214 | + | ATG | 2OG-Fe(II) oxygenase | 2OG-Fe(II) oxygenase | 2.0E-20 | *Phycodnavirus* 1 | [YP_009174691.1](http://www.ncbi.nlm.nih.gov/protein/944325894?report=genbank&log$=prottop&blast_rank=1&RID=ACZR631S01R) |
| 73 | 58,653 | 63,980 | 1775 | + | ATG | gp34: phage tail fiber | hypothetical protein | 1.0E-43 | 11b | [YP_112522.1](http://www.ncbi.nlm.nih.gov/protein/53793622?report=genbank&log$=prottop&blast_rank=1&RID=ACZR631S01R) |
| 74 | 63,977 | 64,384 | 135 | + | ATG | hypothetical protein | hypothetical protein | 2.0E-03 | *Stenotrophomonas panacihumi* | [WP_057645976.1](http://www.ncbi.nlm.nih.gov/protein/951166516?report=genbank&log$=prottop&blast_rank=1&RID=AD4UTJAW015) |
| 75 | 64,388 | 64,858 | 156 | + | ATG | hypothetical protein | hypothetical protein | 2.0E-14 | *Stenotrophomonas maltophilia* | [WP_049447215.1](http://www.ncbi.nlm.nih.gov/protein/896545925?report=genbank&log$=prottop&blast_rank=1&RID=AD4UTJAW015) |
| 76 | 64,891 | 65,058 | 55 | + | ATG | hypothetical protein |  |  |  |  |
| 77 | 65,068 | 65,448 | 126 | + | ATG | hypothetical protein | hypothetical protein | 1.0E-32 | ΦM12 | [YP_009143268.1](http://www.ncbi.nlm.nih.gov/protein/828805907?report=genbank&log$=prottop&blast_rank=1&RID=ACZR631S01R) |
| 78 | 65,450 | 65,956 | 168 | + | ATG | 2OG-Fe(II) oxygenase | 2OG-Fe(II) oxygenase | 2.0E-19 | S-RSM4 | [YP_003097374.1](http://www.ncbi.nlm.nih.gov/protein/255929062?report=genbank&log$=prottop&blast_rank=1&RID=ACZR631S01R) |
| 79 | 65,960 | 66,484 | 174 | + | ATG | hypothetical protein | hypothetical protein | 2.0E-30 | ΦM12 | [YP_009143267.1](http://www.ncbi.nlm.nih.gov/protein/828805906?report=genbank&log$=prottop&blast_rank=1&RID=ACZR631S01R) |
| 80 | 66,481 | 66,849 | 122 | + | ATG | hypothetical protein | hypothetical protein | 3.0E-09 | 1M3-16 | [YP_009037295.1](http://www.ncbi.nlm.nih.gov/protein/643655217?report=genbank&log$=prottop&blast_rank=1&RID=ACZR631S01R) |
| 81 | 66,846 | 67,148 | 100 | + | GTG | hypothetical protein |  |  |  |  |
| 82 | 67,145 | 67,717 | 190 | + | ATG | hypothetical protein | hypothetical protein | 1.0E-14 | HTVC008M | [YP_007518013.1](http://www.ncbi.nlm.nih.gov/protein/460042583?report=genbank&log$=prottop&blast_rank=1&RID=ACZR631S01R) |
| 83 | 67,787 | 68,827 | 346 | + | ATG | gp61: DNA primase | DNA primase | 2.0E-104 | ΦM12 | [YP_009143264.1](http://www.ncbi.nlm.nih.gov/protein/828805903?report=genbank&log$=prottop&blast_rank=1&RID=ACZR631S01R) |
| 84 | 68,824 | 69,006 | 60 | + | ATG | hypothetical protein | hypothetical protein | 6.0E-06 | ACG-2014h | [YP_009008190.1](http://www.ncbi.nlm.nih.gov/protein/589891822?report=genbank&log$=prottop&blast_rank=1&RID=ACZR631S01R) |
| 85 | 68,999 | 69,253 | 84 | + | GTG | hypothetical protein |  |  |  |  |
| 86 | 69,231 | 71,531 | 766 | + | ATG | nrdA | ribonucleotide diphosphate reductase subunit alpha | 0.0E+00 | vB_CsaM_GAP32 | [YP_006987403.1](http://www.ncbi.nlm.nih.gov/protein/414087216?report=genbank&log$=prottop&blast_rank=1&RID=ACZR631S01R) |
| 87 | 71,543 | 72,187 | 214 | + | ATG | methyltransferase | methyltransferase | 4.0E-42 | ΦN3 | [YP_009212249.1](http://www.ncbi.nlm.nih.gov/protein/971758899?report=genbank&log$=prottop&blast_rank=1&RID=ACZR631S01R) |
| 88 | 72,206 | 72,553 | 115 | + | ATG | hypothetical protein | hypothetical protein | 5.0E-07 | ΦCP26F | [YP_007004033.1](http://www.ncbi.nlm.nih.gov/protein/422933993?report=genbank&log$=prottop&blast_rank=1&RID=ACZR631S01R) |
| 89 | 72,553 | 73,599 | 348 | + | ATG | nrdB | ribonucleotide diphosphate reductase subunit beta | 2.0E-128 | vB_CsaM_GAP32 | [YP_006987405.1](http://www.ncbi.nlm.nih.gov/protein/414087218?report=genbank&log$=prottop&blast_rank=1&RID=ACZR631S01R) |
| 90 | 73,596 | 73,829 | 77 | + | ATG | hypothetical protein |  |  |  |  |
| 91 | 73,893 | 74,438 | 181 | + | ATG | RuvC | hypothetical protein | 1.0E-36 | Cr30 | [YP_009098799.1](http://www.ncbi.nlm.nih.gov/protein/725949206?report=genbank&log$=prottop&blast_rank=1&RID=ACZR631S01R) |
| 92 | 74,552 | 74,779 | 75 | + | ATG | hypothetical protein |  |  |  |  |
| 93 | 74,803 | 75,054 | 83 | + | ATG | hypothetical protein |  |  |  |  |
| 94 | 75,054 | 76,184 | 376 | + | ATG | hypothetical protein | hypothetical protein | 5.0E-31 | Cr30 | [YP_009098763.1](http://www.ncbi.nlm.nih.gov/protein/725949170?report=genbank&log$=prottop&blast_rank=1&RID=ACZR631S01R) |
| 95 | 76,184 | 76,414 | 76 | + | ATG | hypothetical protein |  |  |  |  |
| 96 | 76,416 | 76,646 | 76 | + | ATG | hypothetical protein |  |  |  |  |
| 97 | 76,679 | 76,990 | 103 | + | ATG | hypothetical protein |  |  |  |  |
| 98 | 76,987 | 77,865 | 292 | + | GTG | RNase H | Rnase H | 5.0E-83 | S-CBM2 | [AFK66374.1](http://www.ncbi.nlm.nih.gov/protein/388549174?report=genbank&log$=prottop&blast_rank=1&RID=ACZR631S01R) |
| 99 | 77,876 | 78,283 | 135 | + | GTG | T4 Gc 313: hypothetical protein | hypothetical protein | 3.0E-21 | Cr30 | [YP_009099041.1](http://www.ncbi.nlm.nih.gov/protein/725949448?report=genbank&log$=prottop&blast_rank=1&RID=ACZR631S01R) |
| 100 | 78,293 | 78,475 | 60 | + | ATG | hypothetical protein |  |  |  |  |
| 101 | 78,488 | 79,075 | 195 | + | ATG | hypothetical protein | hypothetical protein | 4.0E-34 | ΦN3 | [YP_009212443.1](http://www.ncbi.nlm.nih.gov/protein/971759093?report=genbank&log$=prottop&blast_rank=1&RID=ACZR631S01R) |
| 102 | 79,072 | 79,266 | 64 | + | ATG | hypothetical protein |  |  |  |  |
| 103 | 79,273 | 79,512 | 79 | + | ATG | T4 Gc 321: hypothetical protein | hypothetical protein | 9.0E-17 | ΦM12 | [YP_009143120.1](http://www.ncbi.nlm.nih.gov/protein/828805759?report=genbank&log$=prottop&blast_rank=1&RID=ACZR631S01R) |
| 104 | 79,532 | 80,239 | 235 | + | ATG | PhoH | PhoH | 5.0E-70 | P-SSM3 | [YP_008130050.1](http://www.ncbi.nlm.nih.gov/protein/514231373?report=genbank&log$=prottop&blast_rank=1&RID=ACZR631S01R) |
| 105 | 80,257 | 80,751 | 164 | + | ATG | hypothetical protein | hypothetical protein | 6.0E-40 | CcrColossus | [YP_006988422.1](http://www.ncbi.nlm.nih.gov/protein/414088244?report=genbank&log$=prottop&blast_rank=1&RID=ACZR631S01R) |
| 106 | 80,732 | 81,448 | 238 | + | GTG | exonuclease | hypothetical protein | 7.0E-43 | S-CBM2 | [AFK66381.1](http://www.ncbi.nlm.nih.gov/protein/388549181?report=genbank&log$=prottop&blast_rank=1&RID=ACZR631S01R) |
| 107 | 81,475 | 81,945 | 156 | + | ATG | hypothetical protein | hypothetical protein | 1.0E-09 | Cr30 | [YP_009098845.1](http://www.ncbi.nlm.nih.gov/protein/725949252?report=genbank&log$=prottop&blast_rank=1&RID=ACZR631S01R) |
| 108 | 82,033 | 82,272 | 79 | + | ATG | hypothetical protein | hypothetical protein | 1.0E-06 | uvMED | [BAR35113.1](http://www.ncbi.nlm.nih.gov/protein/787064207?report=genbank&log$=prottop&blast_rank=1&RID=ACZR631S01R) |
| 109 | 82,346 | 82,906 | 186 | + | ATG | SleB | SleB | 1.0E-22 | ΦTE | [YP_007392495.1](http://www.ncbi.nlm.nih.gov/protein/448244775?report=genbank&log$=prottop&blast_rank=1&RID=ACZR631S01R) |
| 110 | 82,910 | 83,398 | 162 | + | ATG | dCMP deaminase | dCMP deaminase | 5.0E-39 | vB_PaeS_PA01_Ab18 | [YP_009125122.1](http://www.ncbi.nlm.nih.gov/protein/764160518?report=genbank&log$=prottop&blast_rank=1&RID=ACZR631S01R) |
| 111 | 83,403 | 83,759 | 118 | + | ATG | deoxycytidylate deaminase | deoxycytidylate deaminase | 2.0E-10 | TMA | [YP_004782245.1](http://www.ncbi.nlm.nih.gov/protein/343960419?report=genbank&log$=prottop&blast_rank=1&RID=ACZR631S01R) |
| 112 | 83,743 | 84,003 | 86 | + | ATG | gp33: hypothetical protein | hypothetical protein | 1.0E-06 | Cr30 | [YP_009099022.1](http://www.ncbi.nlm.nih.gov/protein/725949429?report=genbank&log$=prottop&blast_rank=1&RID=ACZR631S01R) |
| 113 | 84,160 | 84,465 | 101 | + | ATG | hypothetical protein |  |  |  |  |
| 114 | 84,467 | 84,892 | 141 | + | ATG | hypothetical protein |  |  |  |  |
| 115 | 84,892 | 85,305 | 137 | + | ATG | hypothetical protein |  |  |  |  |
| 116 | 85,382 | 85,930 | 182 | + | ATG | hypothetical protein |  |  |  |  |
| 117 | 85,955 | 86,509 | 184 | + | ATG | hypothetical protein |  |  |  |  |
| 118 | 86,519 | 86,731 | 70 | + | ATG | hypothetical protein | hypothetical protein | 7.0E-13 | *Oscillibacter sp.* ER4 | [WP_008981440.1](http://www.ncbi.nlm.nih.gov/protein/496268207?report=genbank&log$=prottop&blast_rank=1&RID=AD4UTJAW015) |
| 119 | 86,737 | 87,144 | 135 | + | ATG | hypothetical protein | hypothetical protein | 1.0E-23 | vB_CsaM_GAP31 | [YP_006986953.1](http://www.ncbi.nlm.nih.gov/protein/414086765?report=genbank&log$=prottop&blast_rank=1&RID=ACZR631S01R) |
| 120 | 87,155 | 87,586 | 143 | + | ATG | hypothetical protein |  |  |  |  |
| 121 | 87,732 | 88,007 | 91 | + | ATG | hypothetical protein |  |  |  |  |
| 122 | 88,009 | 88,188 | 59 | + | ATG | hypothetical protein |  |  |  |  |
| 123 | 88,193 | 88,435 | 80 | + | ATG | hypothetical protein |  |  |  |  |
| 124 | 88,432 | 88,635 | 67 | + | ATG | hypothetical protein |  |  |  |  |
| 125 | 88,632 | 88,772 | 46 | + | ATG | hypothetical protein |  |  |  |  |
| 126 | 88,769 | 89,026 | 85 | + | GTG | hypothetical protein | hypothetical protein | 3.0E-18 | Jay2Jay | [AIW02656.1](http://www.ncbi.nlm.nih.gov/protein/701909128?report=genbank&log$=prottop&blast_rank=1&RID=ACZR631S01R) |
| 127 | 89,023 | 89,274 | 83 | + | ATG | hypothetical protein | hypothetical protein | 6.0E-07 | UFV-P2 | [YP_007518481.1](http://www.ncbi.nlm.nih.gov/protein/496838635?report=genbank&log$=prottop&blast_rank=1&RID=ACZR631S01R) |
| 128 | 89,264 | 89,725 | 153 | + | ATG | hypothetical protein |  |  |  |  |
| 129 | 89,725 | 90,057 | 110 | + | ATG | hypothetical protein |  |  |  |  |
| 130 | 90,050 | 90,364 | 104 | + | ATG | hypothetical protein |  |  |  |  |
| 131 | 90,484 | 91,092 | 202 | + | ATG | (p)ppGpp synthase II | hypothetical protein | 1.0E-34 | *Pseudomonas stutzeri* | [WP_052813543.1](http://www.ncbi.nlm.nih.gov/protein/919374516?report=genbank&log$=prottop&blast_rank=1&RID=AD4UTJAW015) |
| 132 | 91,092 | 91,295 | 67 | + | ATG | hypothetical protein |  |  |  |  |
| 133 | 91,298 | 92,203 | 301 | + | ATG | transposase | transposase | 1.0E-71 | *Pseudomonas mendocina* | [WP_047586214.1](http://www.ncbi.nlm.nih.gov/protein/835617555?report=genbank&log$=prottop&blast_rank=1&RID=AD4UTJAW015) |
| 134 | 92,271 | 92,855 | 194 | + | ATG | hypothetical protein | hypothetical protein | 6.0E-16 | Φ3 | [YP_009207497.1](http://www.ncbi.nlm.nih.gov/protein/971753587?report=genbank&log$=prottop&blast_rank=1&RID=ACZR631S01R) |
| 135 | 93,056 | 93,361 | 101 | + | ATG | hypothetical protein |  |  |  |  |
| 136 | 93,467 | 93,736 | 89 | + | ATG | hypothetical protein |  |  |  |  |
| 137 | 93,746 | 93,976 | 76 | + | ATG | hypothetical protein |  |  |  |  |
| 138 | 94,038 | 95,438 | 466 | + | ATG | hypothetical protein | hypothetical protein | 2.0E-161 | S13 | [YP_009196578.1](http://www.ncbi.nlm.nih.gov/protein/971742082?report=genbank&log$=prottop&blast_rank=1&RID=ACZR631S01R) |
| 139 | 95,658 | 96,224 | 188 | + | ATG | Tk: kinase protein | kinase protein | 5.0E-08 | Cd1 | [ADD21639.1](http://www.ncbi.nlm.nih.gov/protein/289976594?report=genbank&log$=prottop&blast_rank=1&RID=ACZR631S01R) |
| 140 | 96,234 | 96,440 | 68 | + | ATG | hypothetical protein | hypothetical protein | 6.0E-13 | Cr30 | [YP_009098992.1](http://www.ncbi.nlm.nih.gov/protein/725949399?report=genbank&log$=prottop&blast_rank=1&RID=ACZR631S01R) |
| 141 | 96,443 | 97,003 | 186 | + | ATG | hypothetical protein | hypothetical protein | 2.0E-23 | Φpto-bp6g | [YP_009015268.1](http://www.ncbi.nlm.nih.gov/protein/593777424?report=genbank&log$=prottop&blast_rank=1&RID=ACZR631S01R) |
| 142 | 96,993 | 97,181 | 62 | + | ATG | hypothetical protein | hypothetical protein | 8.0E-17 | SFP10 | [YP_004895215.1](http://www.ncbi.nlm.nih.gov/protein/351518487?report=genbank&log$=prottop&blast_rank=1&RID=ACZR631S01R) |
| 143 | 97,159 | 97,410 | 83 | + | ATG | hypothetical protein | hypothetical protein | 9.0E-05 | *Microbacterium sp.* No. 7 | [WP_054686480.1](http://www.ncbi.nlm.nih.gov/protein/938902755?report=genbank&log$=prottop&blast_rank=1&RID=AD4UTJAW015) |
| 144 | 97,403 | 98,062 | 219 | + | ATG | metallophoesterase | metallophoesterase | 1.0E-18 | *Methylomonas denitrificans* | [WP_036280110.1](http://www.ncbi.nlm.nih.gov/protein/738327282?report=genbank&log$=prottop&blast_rank=1&RID=AD4UTJAW015) |
| 145 | 98,136 | 99,215 | 359 | + | ATG | ADP-ribose pyrophosphatase | ADP-ribose pyrophosphatase | 6.0E-112 | IME-SM1 | [AKO61723.1](http://www.ncbi.nlm.nih.gov/protein/857290274?report=genbank&log$=prottop&blast_rank=1&RID=ACZR631S01R) |
| 146 | 99,215 | 99,313 | 32 | + | ATG | hypothetical protein |  |  |  |  |
| 147 | 99,327 | 100,772 | 481 | + | ATG | nicotinamide phosphoribosyltransferase | nicotinamide phosphoribosyltransferase | 4.0E-149 | IME-SM1 | [AKO61724.1](http://www.ncbi.nlm.nih.gov/protein/857290275?report=genbank&log$=prottop&blast_rank=1&RID=ACZR631S01R) |
| 148 | 100,772 | 100,963 | 63 | + | ATG | hypothetical protein |  |  |  |  |
| 149 | 100,960 | 101,154 | 64 | + | GTG | hypothetical protein |  |  |  |  |
| 150 | 101,151 | 101,396 | 81 | + | ATG | hypothetical protein | hypothetical protein | 2.0E-13 | nt-1 | [YP_008125398.1](http://www.ncbi.nlm.nih.gov/protein/514050624?report=genbank&log$=prottop&blast_rank=1&RID=ACZR631S01R) |
| 151 | 101,599 | 101,889 | 96 | + | GTG | hypothetical protein |  |  |  |  |
| 152 | 101,906 | 102,097 | 63 | + | ATG | hypothetical protein |  |  |  |  |
| 153 | 102,104 | 102,313 | 69 | + | ATG | hypothetical protein | hypothetical protein | 2.0E-05 | *Alcanivorax sp.* 43B_GOM-46m | [WP_026949135.1](http://www.ncbi.nlm.nih.gov/protein/652555546?report=genbank&log$=prottop&blast_rank=1&RID=AD4UTJAW015) |
| 154 | 102,610 | 102,834 | 74 | + | ATG | hypothetical protein |  |  |  |  |
| 155 | 103,134 | 103,304 | 56 | + | ATG | hypothetical protein |  |  |  |  |
| 156 | 103,728 | 103,940 | 70 | + | GTG | hypothetical protein | hypothetical protein | 2.0E-05 | *Neptuniibacter caesariensis* | [WP_007019913.1](http://www.ncbi.nlm.nih.gov/protein/494077863?report=genbank&log$=prottop&blast_rank=1&RID=AD4UTJAW015) |
| 157 | 105,246 | 105,767 | 173 | + | ATG | hypothetical protein |  |  |  |  |
| 158 | 106,212 | 106,472 | 86 | + | ATG | hypothetical protein |  |  |  |  |
| 159 | 106,759 | 107,520 | 253 | + | ATG | hypothetical protein | hypothetical protein | 9.0E-14 | *Pseudomonas sp*. Leaf58 | [WP_056798772.1](http://www.ncbi.nlm.nih.gov/protein/948140433?report=genbank&log$=prottop&blast_rank=1&RID=AD4UTJAW015) |
| 160 | 107,664 | 107,888 | 74 | + | ATG | hypothetical protein |  |  |  |  |
| 161 | 108,080 | 108,562 | 160 | + | GTG | hypothetical protein | hypothetical protein | 5.0E-62 | *Candidatus Methylopumilus planktonicus* | [WP_046488394.1](http://www.ncbi.nlm.nih.gov/protein/817115268?report=genbank&log$=prottop&blast_rank=1&RID=AD4UTJAW015) |
| 162 | 111,363 | 111,572 | 69 | + | ATG | hypothetical protein |  |  |  |  |
| 163 | 111,791 | 112,057 | 88 | + | ATG | hypothetical protein |  |  |  |  |
| 164 | 112,831 | 113,079 | 82 | + | ATG | GroES | GroES molecular chaperone protein | 4.0E-03 | *Bradyrhizobium retamae* | [WP_057842828.1](http://www.ncbi.nlm.nih.gov/protein/951471270?report=genbank&log$=prottop&blast_rank=1&RID=AD4UTJAW015) |
| 165 | 113,397 | 113,756 | 119 | + | ATG | hypothetical protein | hypothetical protein | 1.0E-25 | *Flavobacterium filum* | [WP_035655512.1](http://www.ncbi.nlm.nih.gov/protein/737686464?report=genbank&log$=prottop&blast_rank=1&RID=AD4UTJAW015) |
| 166 | 114,007 | 114,354 | 115 | + | ATG | hypothetical protein |  |  |  |  |
| 167 | 114,587 | 114,757 | 56 | + | ATG | hypothetical protein | hypothetical protein | 1.0E-05 | *Nitrosomonas sp.* AL212 | [ADZ27808.1](http://www.ncbi.nlm.nih.gov/protein/325533087?report=genbank&log$=prottop&blast_rank=1&RID=AD4UTJAW015) |
| 168 | 114,750 | 114,974 | 74 | + | ATG | hypothetical protein |  |  |  |  |
| 169 | 115,030 | 115,647 | 205 | + | ATG | hypothetical protein | hypothetical protein | 2.0E-07 | *Hyphomonas adhaerens* | [WP_035570500.1](http://www.ncbi.nlm.nih.gov/protein/737599829?report=genbank&log$=prottop&blast_rank=1&RID=AD4UTJAW015) |
| 170 | 115,647 | 116,264 | 205 | + | ATG | methyltransferase | methyltransferase | 2.0E-12 | uvMED | [BAQ89138.1](http://www.ncbi.nlm.nih.gov/protein/775454967?report=genbank&log$=prottop&blast_rank=1&RID=ACZR631S01R) |
| 171 | 116,266 | 116,916 | 216 | + | ATG | nucleotide-diphospho-sugar transferase | nucleotide-diphospho-sugar transferase | 7.0E-32 | CcrColossus | [YP_006988363.1](http://www.ncbi.nlm.nih.gov/protein/414088185?report=genbank&log$=prottop&blast_rank=1&RID=ACZR631S01R) |
| 172 | 116,909 | 117,700 | 263 | + | GTG | sialyltransferase |  |  |  |  |
| 173 | 117,697 | 117,957 | 86 | + | ATG | nrdC | glutaredoxin | 3.0E-17 | Cr30 | [YP_009098769.1](http://www.ncbi.nlm.nih.gov/protein/725949176?report=genbank&log$=prottop&blast_rank=1&RID=ACZR631S01R) |
| 174 | 117,973 | 118,926 | 317 | + | ATG | glycosyl transferase protein | glycosyl transferase protein | 3.0E-81 | ΦM12 | [YP_009143113.1](http://www.ncbi.nlm.nih.gov/protein/828805752?report=genbank&log$=prottop&blast_rank=1&RID=ACZR631S01R) |
| 175 | 119,098 | 119,577 | 159 | - | ATG | endolysin | endolysin | 2.0E-46 | RL-2015 | [AJG41873.1](http://www.ncbi.nlm.nih.gov/protein/752680733?report=genbank&log$=prottop&blast_rank=1&RID=ACZR631S01R) |
| 176 | 119,690 | 119,914 | 74 | + | ATG | hypothetical protein |  |  |  |  |
| 177 | 119,901 | 120,218 | 105 | + | ATG | hypothetical protein |  |  |  |  |
| 178 | 120,220 | 120,411 | 63 | + | ATG | hypothetical protein |  |  |  |  |
| 179 | 120,428 | 121,342 | 304 | + | ATG | hypothetical protein |  |  |  |  |
| 180 | 121,339 | 121,599 | 86 | + | ATG | hypothetical protein |  |  |  |  |
| 181 | 121,741 | 122,064 | 107 | + | ATG | hypothetical protein |  |  |  |  |
| 182 | 122,068 | 122,268 | 66 | + | ATG | hypothetical protein |  |  |  |  |
| 183 | 122,270 | 122,596 | 108 | + | ATG | hypothetical protein | hypothetical protein | 7.0E-08 | P12053L | [YP_006560897.1](http://www.ncbi.nlm.nih.gov/protein/399528850?report=genbank&log$=prottop&blast_rank=1&RID=ACZR631S01R) |
| 184 | 122,596 | 122,985 | 129 | + | ATG | hypothetical protein | hypothetical protein | 6.0E-18 | *Clostridium botulinum* | [WP_024931995.1](http://www.ncbi.nlm.nih.gov/protein/640493108?report=genbank&log$=prottop&blast_rank=1&RID=AD4UTJAW015) |
| 185 | 123,031 | 123,417 | 128 | + | ATG | hypothetical protein |  |  |  |  |
| 186 | 123,417 | 123,779 | 120 | + | ATG | hypothetical protein | hypothetical protein | 1.0E-27 | *Chryseobacterium sp.* BLS98 | [KMQ60445.1](http://www.ncbi.nlm.nih.gov/protein/861367669?report=genbank&log$=prottop&blast_rank=1&RID=AD4UTJAW015) |
| 187 | 123,786 | 124,307 | 173 | + | ATG | hypothetical protein |  |  |  |  |
| 188 | 124,307 | 124,864 | 185 | + | ATG | hypothetical protein | hypothetical protein | 2.0E-06 | P35 | [YP_001468837.1](http://www.ncbi.nlm.nih.gov/protein/157325418?report=genbank&log$=prottop&blast_rank=1&RID=ACZR631S01R) |
| 189 | 124,839 | 125,438 | 199 | + | ATG | hypothetical protein | hypothetical protein | 3.0E-09 | P35 | [YP_001468837.1](http://www.ncbi.nlm.nih.gov/protein/157325418?report=genbank&log$=prottop&blast_rank=1&RID=ACZR631S01R) |
| 190 | 125,460 | 125,837 | 125 | + | ATG | hypothetical protein | hypothetical protein | 3.0E-26 | ΦM12 | [YP_009143283.1](http://www.ncbi.nlm.nih.gov/protein/828805922?report=genbank&log$=prottop&blast_rank=1&RID=ACZR631S01R) |
| 191 | 125,834 | 125,983 | 49 | + | GTG | hypothetical protein |  |  |  |  |
| 192 | 125,986 | 127,338 | 450 | + | ATG | gp30: DNA ligase | hypothetical protein | 9.0E-117 | *Methylibium petroleiphilum* | [WP_011831629.1](http://www.ncbi.nlm.nih.gov/protein/500156959?report=genbank&log$=prottop&blast_rank=1&RID=AD4UTJAW015) |
| 193 | 127,349 | 127,516 | 55 | + | ATG | hypothetical protein |  |  |  |  |
| 194 | 127,516 | 128,067 | 183 | + | ATG | Fe(II)-dependent oxygenase | Fe(II)-dependent oxygenase | 1.0E-16 | uvMED | [BAQ86661.1](http://www.ncbi.nlm.nih.gov/protein/775452202?report=genbank&log$=prottop&blast_rank=1&RID=ACZR631S01R) |
| 195 | 128,070 | 128,867 | 265 | + | ATG | Ser/Thr phosphatase protein | Ser/Thr phosphatase protein | 5.0E-43 | PBECO_4 | [YP_009150422.1](http://www.ncbi.nlm.nih.gov/protein/849121506?report=genbank&log$=prottop&blast_rank=1&RID=ACZR631S01R) |
| 196 | 128,864 | 129,058 | 64 | + | ATG | hypothetical protein |  |  |  |  |
| 197 | 129,043 | 129,774 | 243 | + | ATG | hypothetical protein | hypothetical protein | 6.0E-69 | Sano | [AHB12055.1](http://www.ncbi.nlm.nih.gov/protein/559198848?report=genbank&log$=prottop&blast_rank=1&RID=ACZR631S01R) |
| 198 | 129,771 | 130,301 | 176 | + | GTG | hypothetical protein |  |  |  |  |
| 199 | 130,330 | 130,521 | 63 | + | ATG | hypothetical protein |  |  |  |  |
| 200 | 130,567 | 131,070 | 167 | + | ATG | hypothetical protein | hypothetical protein | 6.0E-39 | Cr30 | [YP_009098835.1](http://www.ncbi.nlm.nih.gov/protein/725949242?report=genbank&log$=prottop&blast_rank=1&RID=ACZR631S01R) |
| 201 | 131,151 | 131,765 | 204 | + | ATG | gp59: DNA helicase loader | DNA helicase loader | 1.0E-40 | ΦN3 | [YP_009212381.1](http://www.ncbi.nlm.nih.gov/protein/971759031?report=genbank&log$=prottop&blast_rank=1&RID=ACZR631S01R) |
| 202 | 131,859 | 132,815 | 318 | + | ATG | gp32: ssDNA binding protein | ssDNA binding protein | 9.0E-108 | P-SSM2 | [ACY75884.1](http://www.ncbi.nlm.nih.gov/protein/265525087?report=genbank&log$=prottop&blast_rank=1&RID=ACZR631S01R) |
| 203 | 132,883 | 135,045 | 720 | - | ATG | lysozyme | lysozyme | 3.0E-29 | uvMED | [BAR34129.1](http://www.ncbi.nlm.nih.gov/protein/787063052?report=genbank&log$=prottop&blast_rank=1&RID=ACZR631S01R) |
| 204 | 135,049 | 135,495 | 148 | - | ATG | hypothetical protein |  |  |  |  |
| 205 | 135,482 | 135,664 | 60 | - | ATG | gp51: baseplate hub assembly catalyst protein | hypothetical protein | 4.0E-15 | S-SKS1 | [YP_007674616.1](http://www.ncbi.nlm.nih.gov/protein/472341096?report=genbank&log$=prottop&blast_rank=1&RID=ACZR631S01R) |
| 206 | 135,667 | 136,368 | 233 | - | ATG | gp26: baseplate hub subunit protein | hypothetical protein | 3.0E-44 | Cr30 | [YP_009099008.1](http://www.ncbi.nlm.nih.gov/protein/725949415?report=genbank&log$=prottop&blast_rank=1&RID=ACZR631S01R) |
| 207 | 136,377 | 137,273 | 298 | - | ATG | 19.2: hypothetical protein | hypothetical protein | 3.0E-26 | Cr30 | [YP_009099003.1](http://www.ncbi.nlm.nih.gov/protein/725949410?report=genbank&log$=prottop&blast_rank=1&RID=ACZR631S01R) |
| 208 | 137,378 | 137,950 | 190 | + | ATG | endonuclease protein | endonuclease protein | 3.0E-04 | candidate division WS6 bacterium 34_10 | [KUK77195.1](http://www.ncbi.nlm.nih.gov/protein/973122559?report=genbank&log$=prottop&blast_rank=1&RID=AD4UTJAW015) |
| 209 | 137,986 | 138,999 | 337 | - | GTG | Alt | hypothetical protein | 4.0E-05 | *Clostridium sp.* CAG:813 | [CDF00004.1](http://www.ncbi.nlm.nih.gov/protein/524801898?report=genbank&log$=prottop&blast_rank=1&RID=AD4UTJAW015) |
| 210 | 138,996 | 139,613 | 205 | - | ATG | gp2: DNA end protector protein | DNA end protector protein | 3.0E-65 | ΦM12 | [YP_009143037.1](http://www.ncbi.nlm.nih.gov/protein/828805676?report=genbank&log$=prottop&blast_rank=1&RID=ACZR631S01R) |
| 211 | 139,594 | 140,040 | 148 | - | ATG | gp4: head completion protein | head completion protein | 1.0E-51 | P-SSM2 | [YP_214244.1](http://www.ncbi.nlm.nih.gov/protein/61805884?report=genbank&log$=prottop&blast_rank=1&RID=ACZR631S01R) |
| 212 | 140,085 | 140,987 | 300 | + | ATG | gp48: baseplate tail tube cap protein | baseplate tail tube cap protein | 8.0E-18 | ΦEa2809 | [YP_009147536.1](http://www.ncbi.nlm.nih.gov/protein/849120220?report=genbank&log$=prottop&blast_rank=1&RID=ACZR631S01R) |
| 213 | 141,136 | 141,477 | 113 | + | ATG | hypothetical protein | hypothetical protein | 6.0E-06 | *Yersinia pekkaneii* | [WP_049615257.1](http://www.ncbi.nlm.nih.gov/protein/902548615?report=genbank&log$=prottop&blast_rank=1&RID=AD4UTJAW015) |
| 214 | 141,495 | 141,749 | 84 | + | ATG | hypothetical protein |  |  |  |  |
| 215 | 141,755 | 142,057 | 100 | + | ATG | hypothetical protein |  |  |  |  |
| 216 | 142,054 | 142,308 | 84 | + | ATG | hypothetical protein |  |  |  |  |
| 217 | 142,418 | 142,588 | 56 | + | ATG | hypothetical protein |  |  |  |  |
| 218 | 142,588 | 142,962 | 124 | + | ATG | hypothetical protein |  |  |  |  |
| 219 | 142,959 | 143,174 | 71 | + | ATG | hypothetical protein |  |  |  |  |
| 220 | 143,179 | 143,391 | 70 | + | ATG | hypothetical protein |  |  |  |  |
| 221 | 143,486 | 144,034 | 182 | + | ATG | hypothetical protein | hypothetical protein | 4.0E-18 | *Stenotrophomonas maltophilia* | [WP_053451543.1](http://www.ncbi.nlm.nih.gov/protein/923068088?report=genbank&log$=prottop&blast_rank=1&RID=AD4UTJAW015) |
| 222 | 144,036 | 144,227 | 63 | + | ATG | hypothetical protein | hypothetical protein | 3.0E-06 | ΦL7 | [YP_002922638.1](http://www.ncbi.nlm.nih.gov/protein/238695611?report=genbank&log$=prottop&blast_rank=1&RID=ACZR631S01R) |
| 223 | 144,238 | 144,465 | 75 | + | ATG | hypothetical protein |  |  |  |  |
| 224 | 144,462 | 144,728 | 88 | + | ATG | hypothetical protein |  |  |  |  |
| 225 | 144,850 | 145,041 | 63 | + | ATG | hypothetical protein |  |  |  |  |
| 226 | 145,055 | 145,288 | 77 | + | ATG | hypothetical protein |  |  |  |  |
| 227 | 145,285 | 145,521 | 78 | + | GTG | hypothetical protein |  |  |  |  |
| 228 | 145,514 | 145,717 | 67 | + | ATG | hypothetical protein |  |  |  |  |
| 229 | 145,719 | 146,159 | 146 | + | ATG | hypothetical protein |  |  |  |  |
| 230 | 146,447 | 146,971 | 174 | + | ATG | gp53: baseplate wedge subuni | baseplate wedge subunit | 8.0E-35 | Cr30 | YP_009098986.1 |
| 231 | 146,964 | 148,244 | 426 | + | ATG | hypothetical protein | hypothetical protein | 4.0E-13 | RSP15 | [BAU40030.1](http://www.ncbi.nlm.nih.gov/protein/984262034?report=genbank&log$=prottop&blast_rank=1&RID=BB9PXR1W014) |
| 232 | 148,257 | 149,174 | 305 | + | ATG | gp5: baseplate hub and tail lysozyme | baseplate hub and tail lysozyme | 5.0E-45 | uvMED | [BAR35966.1](http://www.ncbi.nlm.nih.gov/protein/787065202?report=genbank&log$=prottop&blast_rank=1&RID=BB9UEDN7014) |
| 233 | 149,207 | 149,608 | 133 | + | ATG | gp25: baseplate wedge subunit | baseplate wedge subunit | 5.0E-30 | uvMED | [BAR29104.1](http://www.ncbi.nlm.nih.gov/protein/787056965?report=genbank&log$=prottop&blast_rank=1&RID=BB9XU1AB015) |
| 234 | 149,610 | 151,400 | 596 | + | ATG | gp6: baseplate wedge protein | baseplate wedge subunit | 2.0E-122 | Cr30 | [YP_009098969.1](http://www.ncbi.nlm.nih.gov/protein/725949376?report=genbank&log$=prottop&blast_rank=1&RID=BBA27K13014) |
| 235 | 151,407 | 155,273 | 1288 | + | ATG | gp7: baseplate wedge subunit protein | baseplate wedge subunit | 4.0E-62 | Cr30 | [YP_009098968.1](http://www.ncbi.nlm.nih.gov/protein/725949375?report=genbank&log$=prottop&blast_rank=1&RID=BBA6X19M014) |
| 236 | 155,314 | 157,041 | 575 | + | ATG | gp8: baseplate wedge subunit protein | baseplate wedge subunit | 6.0E-44 | ΦM12 | [YP_009143024.1](http://www.ncbi.nlm.nih.gov/protein/828805663?report=genbank&log$=prottop&blast_rank=1&RID=BBAARGCE014) |
| 237 | 157,096 | 162,570 | 1824 | + | ATG | VlrC protein | hypothetical protein | 1.0E-122 | Cr30 | [YP_009098966.1](http://www.ncbi.nlm.nih.gov/protein/725949373?report=genbank&log$=prottop&blast_rank=1&RID=BBAERG7B014) |
| 238 | 162,570 | 164,237 | 555 | + | ATG | VlrC protein | VlrC protein | 2.0E-34 | uvMED | [BAR36370.1](http://www.ncbi.nlm.nih.gov/protein/787065669?report=genbank&log$=prottop&blast_rank=1&RID=BBAGMZJ8015) |
| 239 | 164,242 | 164,460 | 72 | + | ATG | hypothetical protein |  | 8.0E-36 |  |  |
| 240 | 164,457 | 165,479 | 340 | + | ATG | hypothetical protein | hypothetical protein | 2.0E-14 | uvMED | [BAQ90538.1](http://www.ncbi.nlm.nih.gov/protein/775456552?report=genbank&log$=prottop&blast_rank=1&RID=BBAP83UA014) |
| 241 | 165,469 | 165,816 | 115 | + | GTG | hypothetical protein | hypothetical protein | 1.0E-04 | MED4-213 | [YP_007673777.1](http://www.ncbi.nlm.nih.gov/protein/472340251?report=genbank&log$=prottop&blast_rank=1&RID=BBAUTG52015) |
