# Supplementary material for: The isolation and characterization of Stenotrophomonas maltophilia T4-like bacteriophage DLP6
Source: PLoS One. 2017 Mar 14;12(3):e0173341. doi: 10.1371/journal.pone.0173341 (PMC5349666; doi:10.1371/journal.pone.0173341)
Supplement: S3 Table — (DOCX) [file pone.0173341.s004.docx]

**Supplementary Table 3. DLP6 tRNA annotations.**

| tRNA | Strand | Start | End | Type | Anticodon | Cove Score |
| --- | --- | --- | --- | --- | --- | --- |
| 1 | + | 92,962 | 93,035 | Arg | CCT | 61.34 |
| 2 | + | 103,314 | 103,387 | Pro | TGG | 73.92 |
| 3 | + | 103,398 | 103,469 | Ile | GAT | 43.6 |
| 4 | + | 103,950 | 104,034 | Leu | TAG | 55.46 |
| 5 | + | 104,297 | 104,368 | Gly | GCC | 74.95 |
| 6 | + | 104,656 | 104,729 | Leu | CAA | 50.06 |
| 7 | + | 104,738 | 104,829 | Ile | TAT | 46.18 |
| 8 | + | 104,845 | 104,917 | Phe | GAA | 44.78 |
| 9 | + | 106,026 | 106,099 | Arg | CCG | 64.25 |
| 10 | + | 106,481 | 106,557 | Thr | CGT | 68.07 |
| 11 | + | 107,530 | 107,612 | Pseudo | GTT | 29 |
| 12 | + | 107,978 | 108,050 | Lys | TTT | 55.23 |
| 13 | + | 109,099 | 109,169 | Ile | GAT | 63.46 |
| 14 | + | 109,728 | 109,799 | Lys | CTT | 56.9 |
| 15 | + | 110,017 | 110,089 | Ala | TGC | 68.8 |
| 16 | + | 110,184 | 110,255 | Cys | GCA | 47.48 |
| 17 | + | 110,348 | 110,418 | Gln | CTG | 63.37 |
| 18 | + | 110,432 | 110,504 | Trp | CCA | 66.76 |
| 19 | + | 110,516 | 110,587 | Val | TAC | 39.72 |
| 20 | + | 110,832 | 110,903 | Thr | GGT | 74.72 |
| 21 | + | 111,078 | 111,149 | Val | GAC | 67.9 |
| 22 | + | 111,579 | 111,652 | Arg | TCT | 56.91 |
| 23 | + | 112,066 | 112,139 | Leu | GAG | 56.11 |
| 24 | + | 112,149 | 112,221 | Phe | GAA | 74.14 |
| 25 | + | 112,574 | 112,645 | His | GTG | 44.49 |
| 26 | + | 112,656 | 112,728 | Lys | CTT | 75.8 |
| 27 | + | 112,740 | 112,813 | Arg | ACG | 69.71 |
| 28 | + | 113,092 | 113,166 | Pro | CGG | 65.21 |
| 29 | + | 113,330 | 113,403 | Leu | CAG | 56.95 |
| 30 | + | 113,763 | 113,834 | Sup | CTA | 48.3 |
